# A periplasmic layer formed by an outer membrane lipoprotein governs the cell-envelope integrity and stiffness of *Leptospira interrogans*

**DOI:** 10.1038/s42003-026-10443-1

**Published:** 2026-06-04

**Authors:** Keigo Abe, Nobuo Koizumi, Hiroko Takazaki, Mika Hirose, Kyosuke Takabe, Takayuki Kato, Shuichi Nakamura

**Affiliations:** 1 Department of Applied Physics, Graduate School of Engineering, Tohoku University, Sendai, Miyagi Japan; 2 Department of Bacteriology I, National Institute of Infectious Diseases, Japan Institute for Health Security, Shinjuku, Tokyo Japan; 3 Institute for Protein Research, The University of Osaka, Suita, Osaka Japan

**Keywords:** Bacterial structural biology, Bacteriology, Biophysics

## Abstract

The structural integrity and mechanical properties of the cell envelope are crucial for bacterial physiology and dynamics; however, how specific molecular components govern these properties remains poorly understood. Here, we report the structural and mechanical functions of LipL32, the most abundant outer-membrane (OM) lipoprotein in the pathogenic spirochete *Leptospira interrogans*. LipL32 accounts for approximately 75% of the total OM proteins, yet its functional role remains enigmatic. Cryo-electron microscopy reveals that the highly dense LipL32 forms a thin layer in the periplasmic space, positioned adjacent to the peptidoglycan layer. The structural analysis also demonstrates that the disruption of *lipL32* by transposon insertion results in irregular deformation of OM and spatial variability in the width of the periplasmic space. Quantitative measurements by optical tweezers confirm that the *lipL32::*Tn mutant exhibits a significant reduction in whole-cell stiffness. Consistent with this mechanical impairment, the *lipL32::*Tn mutant displays a diminished ability to penetrate a heterogeneous agar mesh model that mimics the host’s extracellular matrix, suggesting an important role for cell rigidity in initial infection via damaged skin. This work provides a mechanobiological insight into how lipoproteins contribute to cell-envelope integrity as a stabilizer, conferring stiffness and invasiveness on bacterial pathogens.

## Abstract (web summary)

Abe et al. show that LipL32, the most abundant outer-membrane (OM) lipoprotein in the pathogenic spirochete Leptospira interrogans, forms a previously unrecognized periplasmic layer that maintains outer membrane integrity. Its disruption reduces cellular stiffness and impairs matrix penetration, underscoring its key role in bacterial rigidity and invasion.

## Introduction

The cell envelope of Gram-negative bacteria is more than a passive barrier; it is a complex, multi-functional organelle that governs the cellular mechanical properties and physiology^1^, and is associated with various dynamics such as motility^2^. Regarding the outer membrane (OM), its role as a permeability barrier is well-established, but recent studies revealed a variety of functions heretofore unknown. For instance, though the peptidoglycan (PG) has been recognized as a major determinant for cell shape, OM is also involved in the improvement of bacterial cell shape through modification by the enhanced synthesis of lipopolysaccharides (LPS)^3^. Also, the connection between the OM and PG via a small α-helical OM lipoprotein, called Lpp (also known as Braun’s lipoprotein), confers resistance against osmotic shock on *Escherichia coli*^4^.

Among Gram-negative bacteria, spirochetes, including pathogens such as *Borrelia burgdorferi* (Lyme disease), *Treponema pallidum* (syphilis), *Brachyspira hyodysenteriae* (swine dysentery), and *Leptospira interrogans* (leptospirosis), are structurally distinguished by the presence of periplasmic flagella (PFs) situated between the OM and PG layer^5^. While the core machinery for OM biogenesis and protein secretion in spirochetes is largely conserved with other Gram-negative bacteria, their OM components are enriched with diverse proteins involved in host interactions and immune evasion^6^. For instance, OspA is a major immunogenic lipoprotein in *B. burgdorferi*^7^. DbpA and DbpB of *B. burgdorferi* function as adhesins with binding affinity for the extracellular matrix (ECM) component decorin^8^, while *T. pallidum* Tp0751 exhibits affinity for laminin^9^.

This study focuses on the OM proteins of pathogenic *Leptospira*, the causative agent of leptospirosis. Leptospirosis is a worldwide zoonosis, exhibiting an extremely wide range of host spectra^10^. *Leptospira* spp. possesses two short PFs (one flagellum per cell end) in the short-pitch helical cell body and moves in liquid and on surfaces^5,11^. *Leptospira* spp. comprises pathogenic and nonpathogenic (saprophytic) species. The pathogenic strains are colonized in the proximal renal tubules of maintenance hosts, typically rodents in nature, and shed into the environment upon urination. Contact with the urine of maintenance hosts or contaminated soil and water is an opportunity for percutaneous infection of animals and humans, resulting in a range of symptoms, such as fever, headache, jaundice, and multi-organ failure^10,12^. Even limited to human cases, leptospirosis causes over 60,000 deaths annually worldwide, primarily in tropical regions^13^, thus posing a public health problem requiring early address.

Vaccination is a key strategy for preventing the dissemination of leptospirosis; however, currently available vaccines are serovar-specific inactivated vaccines targeting LPS. Because *Leptospira* spp. are classified into more than 300 serovars based on LPS structure, the identification of broadly protective vaccine targets remains an important challenge. In addition to LPS, *Leptospira* spp. contains diverse protein components, some of which are conserved among pathogenic species and have therefore been investigated as candidates for the development of more broadly protective vaccines. For example, immunization by combining two OM proteins, LipL41 and OmpL1, provides a protective effect^14^. Immunogenic properties of LigA and LigB, containing immunoglobulin-like repeats, are also confirmed in mice^15^. LipL32, assayed in this study, is commonly carried by pathogenic species and accounts for ~75% of the total OM protein^16^. LipL32 is not exposed to the membrane surface^17^, and immunization by the protein does not induce a protective immune response^18^. Although the lipoprotein unique to pathogens has been expected to be a crucial virulence factor, the mutant lacking LipL32 retains pathogenicity, causing an acute manifestation comparable to that of the parental strain^19^. Thus, despite being a pathogen-specific protein, LipL32 is not believed to be an essential virulence factor, and its role remains unclear.

In this study, we sought to elucidate the significance of LipL32 by determining its subcellular location in situ using cryo-electron microscopy (cryo-EM) and characterizing the effect of LipL32 deficiency on the bacterial physical properties using biophysical experiments. Our experiments revealed a mechanism by which an abundant but non-essential lipoprotein contributes to the cell envelope integrity and cell-body stiffness of *L. interrogans*. We also bridge the structural and mechanical functions of LipL32 to spirochete infection.

## Results

### LipL32 forms a thin layer and defines the periplasmic width

**Fig. 1:**
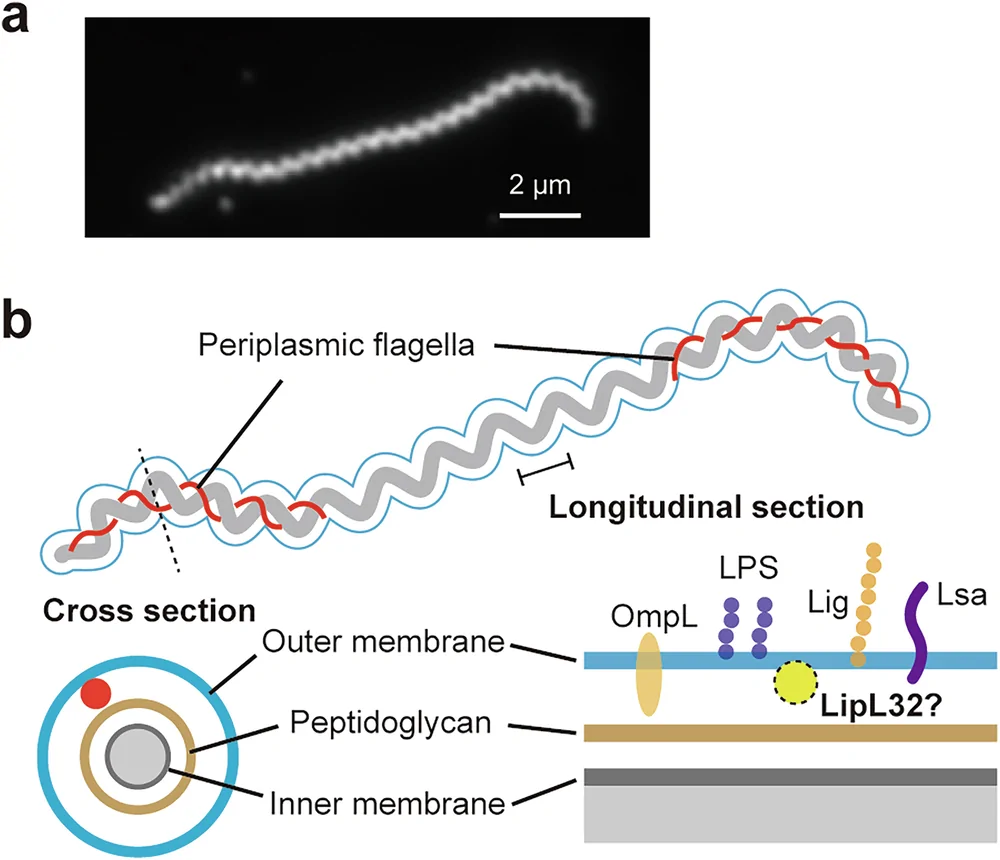
Morphology and structure of *Leptospira interrogans.* **a** A dark-field micrograph of *L. interrogans*. Periplasmic flagella curve the cell ends, and the entire cell body exhibits a short-pitch helix. **b** Schematic diagrams of the whole cell and membrane structures. Short periplasmic flagella (red) extend from both cell ends. The cross-section and longitudinal-section views illustrate the membrane structure typical of Gram-negative bacteria. Representative outer-membrane components are indicated in the longitudinal-section view. LipL32 is known not to be exposed to the outer-membrane surface, but its subcellular location is the subject of this study.

**Fig. 2:**
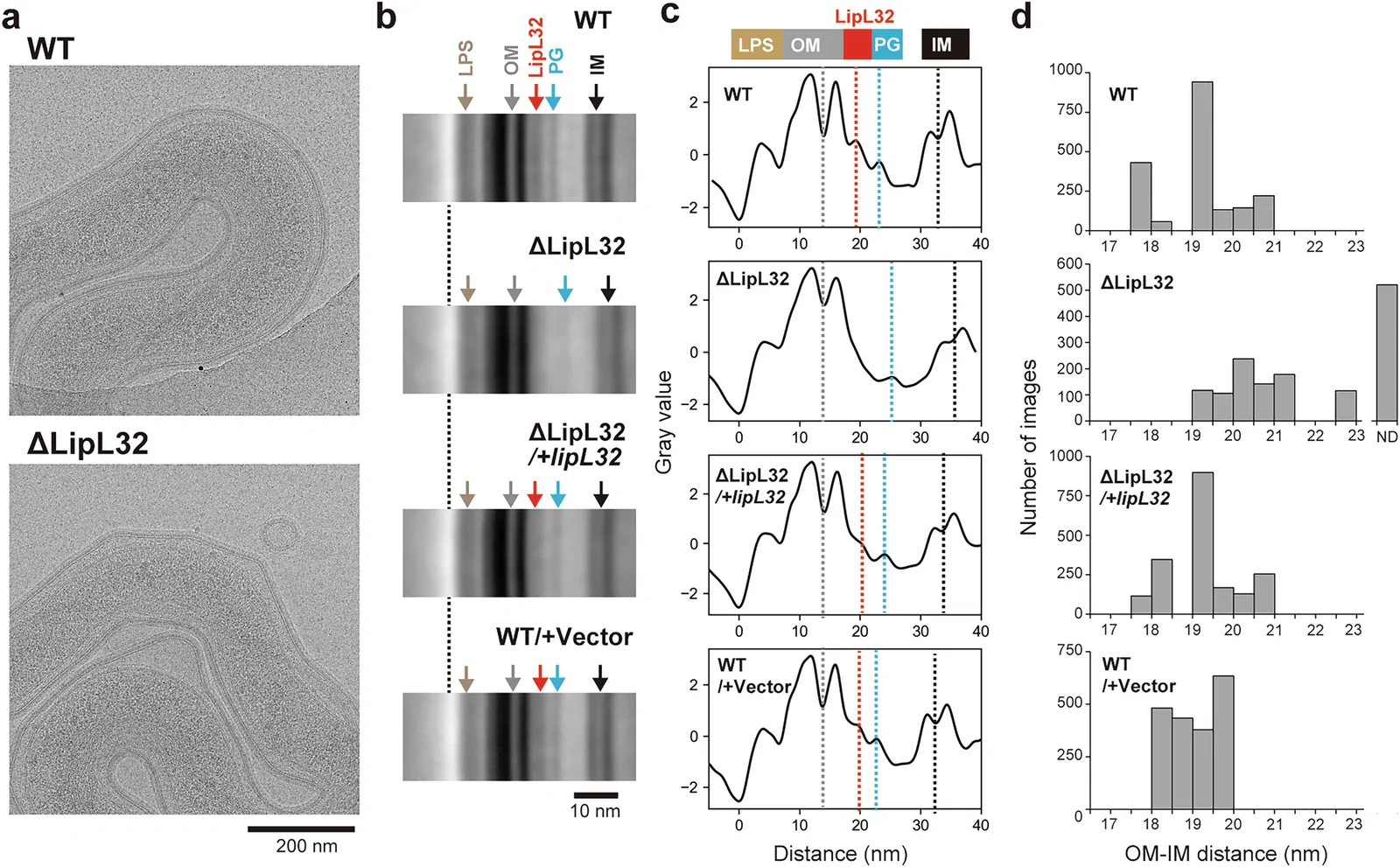
Effect of ΔLipL32 on the membrane structure. **a** Cryo-electron micrographs of the WT and ΔLipL32 cells. **b** Enlarged average images of the cellular envelope comparing WT, ΔLipL32, the complemented strain (ΔLipL32/+*lipL32*), and the vector control (WT/+Vector). The images are aligned by matching the outer position of the LPS layer (vertical dashed lines). The membrane layers are indicated: outer membrane (OM), peptidoglycan layer (PG), and inner membrane (IM). Note the appearance of a distinct inner OM layer (red arrows) present only in the WT, the complemented strain, and the vector control. The results of 2D clustering analysis are shown in Supplementary Fig. 3. **c** Averaged density profiles across the cell envelope. **d** Distribution of the OM-IM distance (periplasmic width). ND (No Data) category accounts for images where the IM was undetectable within the ROI (i.e., distance > 25 nm) or where IM density was averaged out during image averaging due to positional instability of IM.

As shown in Fig. 1, *L. interrogans* possesses a spiral-shaped cell body with two flagella located immediately beneath the OM. The OM contains a diverse array of proteins, including many with unknown functions, among which LipL32 is the most abundant pathogen-specific lipoprotein. An immunofluorescent assay with an anti-LipL32 antibody showed that LipL32 was detected in *L. interrogans* cells whose OM was permeabilized with methanol^17^. This demonstrated that LipL32 is not exposed on the OM surface, but the precise subcellular location of LipL32 in the leptospiral cell has been elusive. To identify the subcellular localization of LipL32 in the wild-type (WT) *L. interrogans*, we used a *lipL32*-disrupted mutant generated by random transposon insertion (*lipL32::*Tn, hereafter ΔLipL32) as a control (Supplementary Fig. 1) and compared their membrane structures by cryo-EM. Disruption of *lipL32* did not affect the growth rate and cell morphology observed by dark-field microscopy (Supplementary Fig. 2), but cryo-EM showed irregular deformation in the OM (Fig. 2a). We found an apparent layered density in the inner part of OM facing PG in the WT cells, which vanished in the ΔLipL32 strain and was confirmed in its *lipL32*-complemented strain and the WT carrying an empty vector (Fig. 2b). This observation suggests that LipL32 is localized on the periplasmic side of the OM, possibly via insertion of its lipid moiety into the membrane, forming a thin layer immediately beneath the OM.

We also found that the distance between the OM and IM of the ΔLipL32 mutant was wider than that of strains expressing LipL32 (Fig. 2c, d). Notably, the IM could not be detected in about 40% of the analyzed images of the ΔLipL32 mutant (represented as ND in Fig. 2d). In addition, both the position and density of the PG layer were also altered in the ΔLipL32 mutant (Fig. 2c and Supplementary Fig. 4). These IM-undetectable images suggest that the IM is not fixed at a uniform position but varies between cells (Supplementary Fig. 5), leading to a reduction in signal intensity below the detection threshold due to image averaging, or that the IM is located beyond the measurable range (i.e., OM-IM distance > 25 nm).

### Cell stiffness

**Fig. 3:**
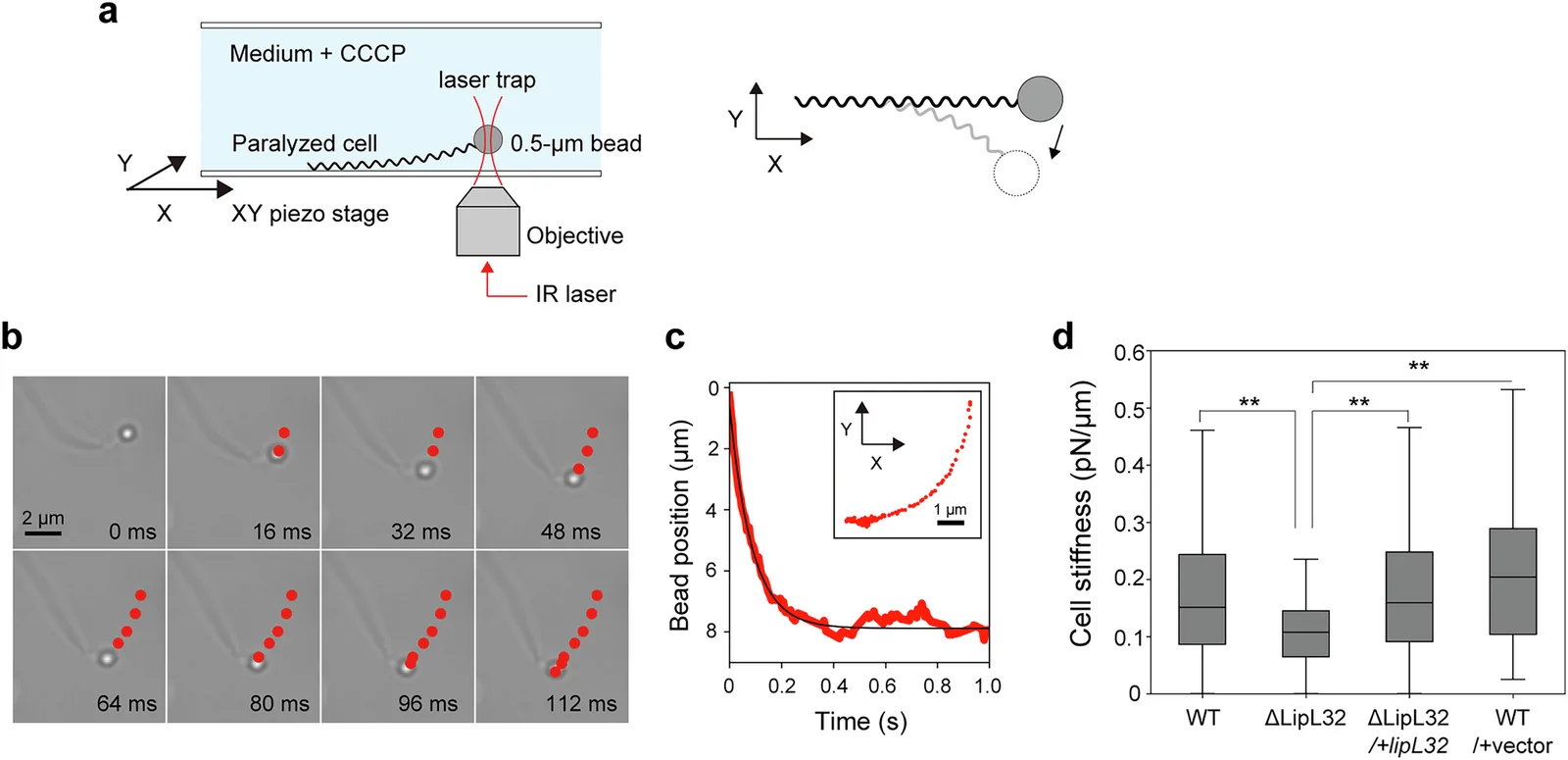
Effect of ΔLipL32 on the cell stiffness. **a** Schematic of the optical tweezers setup used for measuring cell stiffness (left panel). The right schematic illustrates cell bending by moving the piezo stage in the X-Y plane. **b** Example of bead position recovery after release from the laser trap. At 0 ms, the bead attached to the cell end is trapped by a laser. Red dots indicate the bead positions. The example shows two-dimensional movement, but 3D trajectory analysis was performed for all measurements (details in the Supplementary Fig. 6). **c** Time-dependence of bead displacement after release (red line). The black line is the result of exponential fitting by $A\exp \left[(-k/\gamma )t\right]+C$, where k is the spring constant (measured as a cell stiffness in this study), γ is the drag coefficient, and A and C are arbitrary constants included in the fitting function (see “Materials and Methods for details). The trajectory of the bead positions is shown in the inset. **d** Cell stiffness values for WT (*n* = 60 cells), ΔLipL32 (*n* = 49 cells), ΔLipL32/+*lipL32* (*n* = 56 cells), and a vector control (*n* = 34 cells). In the box plots, the center line indicates the median, the box limits represent the first and third quartiles (25th and 75th percentiles), and the whiskers extend to the minimum and maximum values excluding outliers (calculated as 1.5× interquartile range). Statistical analysis was performed by the two-sided Mann–Whitney *U* test (**P < 0.01): WT vs ΔLipL32 *P* = 0.0070, WT vs ΔLipL32/+*lipL32 P* = 0.8967, WT vs vector control *P* = 0.2152, ΔLipL32 vs ΔLipL32/+*lipL32 P* = 0.0023, ΔLipL32 vs vector control *P* = 0.0003, and ΔLipL32/+*lipL32* vs vector control *P* = 0.2775.

The structural evidence yielded by the cryo-EM implied the effect of *lipL32-*disruption on the mechanical properties of the cell membrane due to the loss of the LipL32-layer and the disruption of membrane integrity (Fig. 2). Therefore, we compared the rigidity of the WT and ΔLipL32 strains by measuring individual cells using optical tweezers. Among the cells adhering to the glass surface, cells whose bead-adhered cell ends were apart from the glass surface were selected, and the beads were trapped with an infrared laser (Fig. 3a). The cell was bent by moving the microscope stage to change the trapped bead position, and then the recovery of the bead position (cell morphology) to the original position after releasing from the laser trap was traced using machine-learning combined image analysis (Fig. 3b and Supplementary Fig. 6). The cell rigidity was estimated as a spring constant in this experiment, computed from the recovery rate of the bead position and drag coefficient acting on the bead (Fig. 3c). The stiffness measurement revealed that ΔLipL32 reduced the cell stiffness by half of the WT: 0.19 ± 0.02 pN/µm for WT and 0.11 ± 0.01 pN/µm for ΔLipL32. The reduced rigidity of ΔLipL32 was recovered by the complementation of the *lipL32* gene up to the WT level (0.18 ± 0.02 pN/µm). Thus, an anomaly in the cell-envelope integrity significantly reduces the cell strength.

### Penetration into a heterogeneous gel

**Fig. 4:**
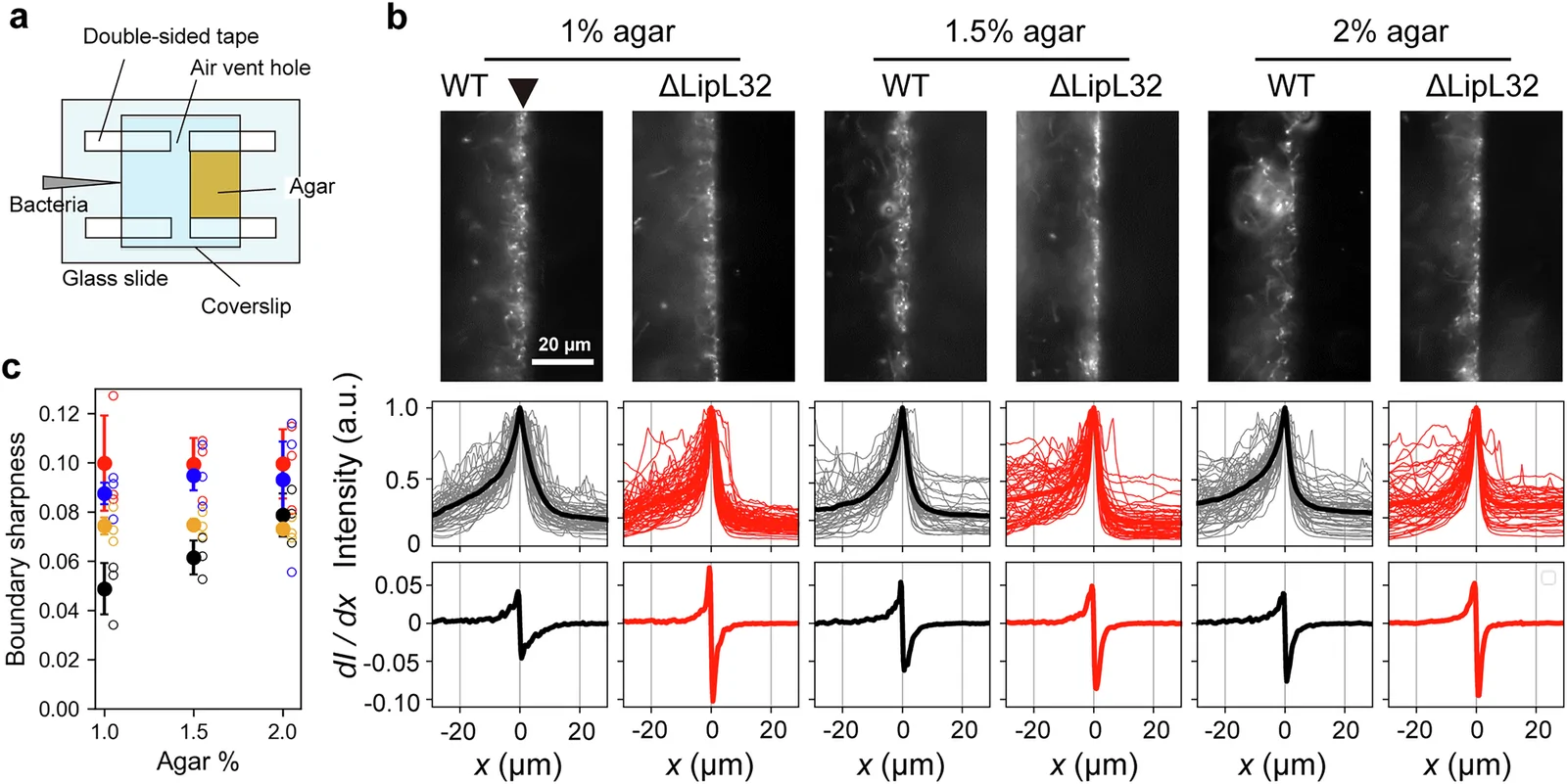
Bacterial penetration into a heterogeneous gel. **a** A flow chamber containing a liquid-agar boundary. **b**, top Micrographs of liquid-gel interfaces exposed with the WT and ΔLipL32 cells, examined in the presence of 1, 1.5, and 2% agar. The black triangle shown in the micrograph of WT in 1% agar indicates the boundary of the liquid containing bacteria and agar. The middle panels show brightness profiles around the liquid-gel boundaries (results of ΔLipL32/+*lipL32* and vector control are shown in Supplementary Fig. 7). Thin lines represent individual profiles from independent measurements, and the thick line indicates the average of these profiles. The spatial gradient of brightness (bottom), ${dI}/{dx}$, was determined by differentiating the brightness profiles. Larger values correspond to sharper boundaries. **c** Dependence of the boundary sharpness on the agar concentration: Black for WT, red for ΔLipL32, blue for ΔLipL32/+*lipL32*, and orange for a vector control. The maximum values of ${dI}/{dx}$ were defined as the boundary sharpness. Average values and standard deviations of three independent experiments (*n* = 3) are shown. Individual data from each experiment are shown as open circles.

The major portal of entry for *L. interrogans* is thought to be injured skin that reaches the dermis^10,12^. Given this infection route and our optical tweezer data, we hypothesized that reduced stiffness caused by the loss of LipL32 would impair penetration into host tissue. To test this, we compared the penetration ability of WT and ΔLipL32 mutant strains into agar gels with heterogeneous mesh structures resembling dermis (Fig. 4a). The substrate modulus of skin dermis is 20–50 kPa^20^, which is a very similar range of 1–2% agar^21^. The pore size of 1% agar is 100–300 nm^22^, comparable to the *L. interrogans* cell size (~150 nm in width), and it decreases with the agar concentration^23^. Therefore, this in vitro assay allows us to qualitatively evaluate the mechanical performance of the *L. interrogans* penetration.

The penetration assay showed that WT cells disrupted the gel–liquid interface, creating rough boundaries due to bacterial entry. In contrast, ΔLipL32 mutants attached to the gel surface but failed to penetrate deeply, leaving a sharp liquid–gel interface intact (Fig. 4b). The sharpness of the liquid-agar boundary exposed to the WT cells was increased with the agar concentration, meaning impairment of bacterial penetration at high agar concentrations (Fig. 4c). These results suggest that a certain level of cell stiffness is critically required for efficient penetration into the dense tissue-mimicking matrix. (Results of the *lipL32*-complemented and vector-control strains are discussed below).

## Discussion

This study sought to elucidate the enigmatic role of LipL32, the most abundant OM lipoprotein in the zoonotic spirochete *L. interrogans*. Despite its specific presence in pathogenic species, its precise function and necessity for virulence have been debated. Using cryo-EM and biophysical experiments, we have provided structural and functional evidence demonstrating that LipL32 forms a distinct layer in the periplasmic space (PS) that acts as a critical mechanical stabilizer for the cell envelope and confers strength on the cell body. The reduced cell stiffness by the loss of LipL32 affects penetration into tissue-mimicking matrices.

**Fig. 5:**
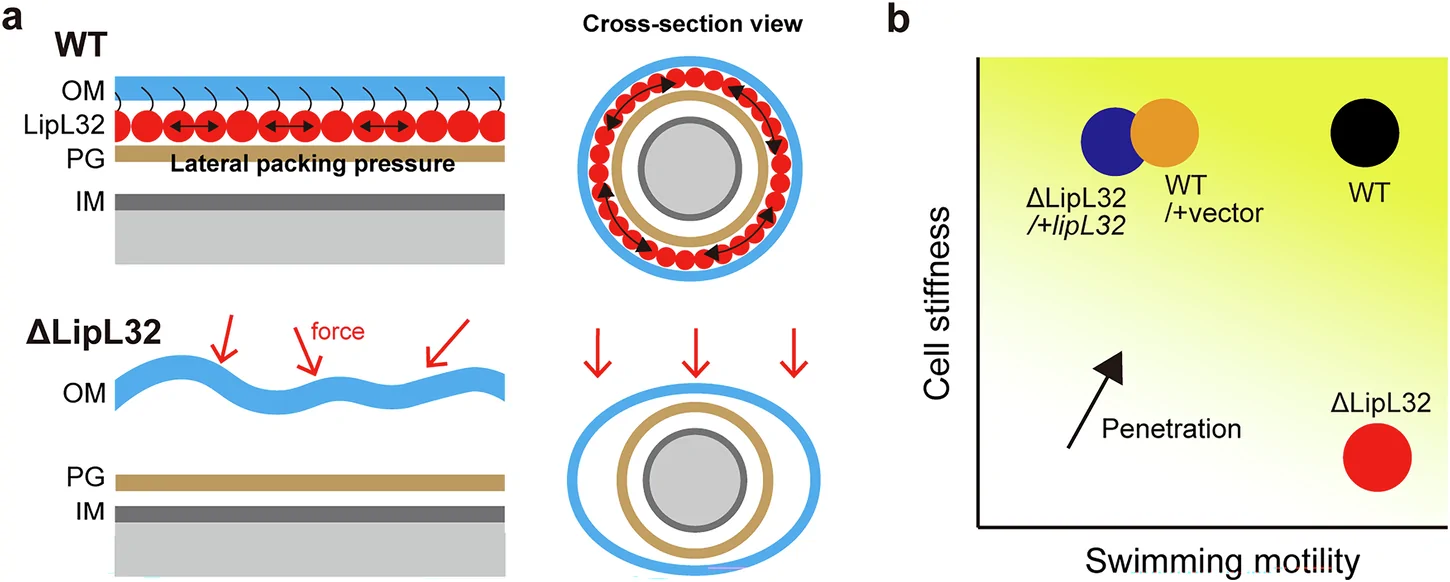
Proposed model for the structural role of LipL32 and association with the penetration ability. **a** LipL32 proteins (red spheres, upper panel) form a distinct, highly dense layer within the periplasmic space of *L. interrogans*. This dense packing is hypothesized to generate lateral packing pressure (black arrows), which acts as a structural scaffold to reinforce the outer membrane (OM). In the absence of the LipL32 layer (lower panel), the cell envelope becomes mechanically fragile and susceptible to external forces—such as the surface tension encountered during cryo-EM sample preparation—leading to the irregular membrane deformation and non-uniform periplasmic width observed in this study. The left schematics illustrate the cross-sectional view of the leptospiral cell body. **b** A reduction of the cell stiffness interferes with the bacterial penetration into gel-like structures, even while fully maintaining motility, vice versa. The thicker yellow in the background represents higher penetration ability.

Our most striking finding is the cryo-EM visualization of a layered density formed by the highly abundant LipL32 immediately beneath the OM in the PS (Fig. 2b, c). Our results revealed an unexpected arrangement where this massive amount of protein is tightly packed to form a structural layer that is distinct from the conventional three-membrane structure of Gram-negative bacteria, OM, PG, and IM (Fig. 5a, upper panel). Although LipL32 molecules may lack the lateral-bonding characteristic of an actual biological membrane, their dense organization could function as a pseudo-structural scaffold stabilized by lateral packing pressure (black arrows in Fig. 5a, upper panel). While cryo-EM showed irregular deformation of the cell envelope upon *lipL32* disruption (Fig. 2a, lower panel), such structural defects were not observed by dark-field microscopy (Supplementary Fig. 2). Most likely, the membranes of the ΔLipL32 mutant, which had been fragilized by the loss of LipL32 periplasmic layer, suffered more severe damage by external forces such as rapid water absorption during the cryo-EM sample preparation process than those of WT. This may explain the increased OM-IM distance observed by cryo-EM (Fig. 5a, lower panel), leading to the result that IM was not detected in nearly 40% of the ΔLipL32 strain data (Fig. 2d). Meanwhile, the fact that non-pathogenic *Leptospira* species synthesize PG normally without LipL32^24^ yields the hypothesis that the LipL32 layer is a pathogen-specific evolutionary enhancement to provide extra mechanical rigidity beyond that required for basic viability. Furthermore, while our binary comparison between WT and the ΔLipL32 mutant highlights the structural contribution of LipL32, it does not address how variations in protein density may influence mechanical properties. Future studies that systematically modulate LipL32 expression levels will be required to directly test the proposed density–mechanics relationship and to further refine the “lateral pressure” model.

While lateral packing pressure provides a feasible mechanism for OM reinforcement, the possibility of LipL32-PG interaction cannot be excluded, especially given that another OM lipoprotein, LipL21, is known to bind PG^25^. Furthermore, elasticity measurement using an atomic-force microscope showed that the loss of a penicillin-binding protein (PBP) in *Staphylococcus aureus* reduces the cell rigidity^26^. Although LipL32 lacks a typical PGB motif, assuming the direct interaction between LipL32 and PG allows for an alternative model. The structural demands of spirochetal membranes, which undergo constant cyclic deformation, evoke a functional similarity to eukaryotic axonemes^27^. In the system, nexin linkers maintain the relative positioning of adjacent microtubule doublets and resist excessive shear deformation^28^. By analogy, we hypothesize that the dense LipL32 layer functions as a structural tether that bridges the OM and PG, providing the necessary restoring force to stabilize the periplasmic architecture. High-density packing of LipL32 may realize multi-point physical interactions with the PG layer, acting as a mechanical spring that provides a restoring force against external stresses, such as the surface tension encountered during cryo-EM sample preparation. Whether and how LipL32 physically interacts with the PG layer remains an open question. Direct biochemical evidence for LipL32–LipL32 or LipL32–PG interactions is currently lacking, and future studies employing complementary approaches, such as co-immunoprecipitation or in vitro binding assays using purified LipL32 and PG sacculi, will be required to validate this model. Elucidating these molecular mechanisms will be an important direction for future research.

As expected from the structural evidence, the loss of the LipL32 layer and the resulting periplasmic instability are translated directly into a significant loss of cell rigidity (Fig. 3). This demonstrates a direct link between the LipL32 layer and the overall mechanical robustness of the spirochete cell. The necessity of this rigidity was confirmed by the penetration assay (Fig. 4). The ΔlipL32 mutant, which is significantly softer, failed to penetrate the dense tissue-mimicking agar matrix, whereas WT cells effectively disrupted the gel interface. It has been suggested that the reduced cell rigidity of *S. aureus* due to PBP deficiency possibly affects the bacterial invasion into the osteocyte network^29^. More broadly, cell mechanical properties are increasingly recognized as critical determinants of cellular function and invasiveness. For example, alterations in cell stiffness are known to impair capillary passage in malaria-infected red blood cells, highlighting the importance of appropriate mechanical flexibility for successful tissue traversal^30^. Our results establish that, in addition to the known essentiality of motility, sufficient cell rigidity could be a critical and separable factor required for *L. interrogans* to penetrate dense, heterogeneous structures that mimic host dermal tissue.

While our primary data support the necessity of cell stiffness, the penetration assay revealed complexity: the complemented and vector control strains, despite exhibiting the WT-level stiffness (Fig. 3d), did not fully recover their penetration rates (Fig. 4c). As detailed in the Supplementary Information, we found that these mutants carrying plasmids slowed the swimming speed despite no impact on cell-body rotation (Supplementary Fig. 8). The swimming-dependent locomotion of spirochetes, including *Leptospira*, is achieved by coordinated rotation of PFs residing at both ends of the cell body. The mechanism of PF coordination is elusive, but it is believed to involve the sensory and signal transduction, such as the chemotaxis system^5,6^. The genetic manipulation or antibiotics added to maintain the plasmids in the mutants might affect gene regulation and protein dynamics, associated with the coordinated PF rotation, independently of the structural role of LipL32. This observation is significant, as it suggests that both sufficient cell rigidity (mediated by LipL32) and efficient motility (mediated by PF coordination) are independent yet crucial determinants for optimal invasion to gel structure (Fig. 5b). This interpretation could reconcile our findings with previous animal studies that showed the ΔLipL32 mutant retains pathogenicity when inoculated into the eye and intraperitoneally^19^. We hypothesize that the structural role of LipL32, providing mechanical strength, is specifically essential for navigating the physical challenges posed by the dense ECM of the damaged skin dermis, which is the major natural route of infection. While the measured motility parameters remained unaffected by the loss of the LipL32 layer (Supplementary Fig. 8), the mechanical challenges at the liquid-gel interface are likely more severe. The structural instability and non-uniform periplasmic width observed in ΔLipL32 may render the cell envelope susceptible to irregular deformation upon initial contact with the agar surface. Such localized compression of the PS could potentially interfere with flagellar movement or the spatial arrangement of PFs at the cell poles. In contrast, infection via less physically restrictive routes (e.g., mucosal route such as the eye) may bypass the need for this enhanced rigidity.

In *E. coli*, the most abundant protein is Lpp, which exists at approximately 10^6^ copies per cell^31^. Lpp functions as a “tether” that covalently anchors the OM to the PG layer. The periplasmic distance between the OM and PG is strictly determined by the length of the α-helix comprising Lpp, and this spacing even correlates with the length of the flagellar rod spanning the envelope^32^. Lpp is involved in OM integrity through interactions with other OM components, such as Pal and OmpA^33^, while also having a responsibility for defining the cellular mechanical strength^34,35^. In this regard, LipL32 and Lpp share functional similarities as key players for determining cell stiffness and envelope stability. However, while covalent bridging, as seen in Lpp, appears highly effective for structural reinforcement, LipL32 lacks a typical PGB motif as mentioned above. If LipL32 indeed lacks a covalent anchoring mechanism, what is the evolutionary significance of such a dense yet non-covalent layer? We hypothesized that this “quasi-rigid” architecture is intrinsically linked to the unique motility of *L. interrogans*. *L. interrogans* moves by rotating PFs, causing high-speed and dynamic deformations of the entire cell body^5,36^. Furthermore, during crawling motility on host tissue surfaces, the spirochetes undergo even more pronounced deformations compared to swimming in liquid^37^. In the context of Lpp, the softening of *E. coli* cells by the Lpp-related mutation increases the sensitivity against antibiotics^34^, suggesting the significance of the cell stiffness given by the envelope integrity for bacterial fitness and behaviors. Instead, we speculate that *L. interrogans* evolved a highly dense protein layer rather than rigid covalent anchors to achieve a unique mechanical profile. By utilizing the strong lateral packing pressure generated by LipL32, the cell might maintain the necessary rigidity for tissue penetration while retaining the spring-like elasticity required for its sophisticated and dynamic locomotion.

In conclusion, our study defines LipL32 as a critical periplasmic structural element that provides the mechanical stability necessary for *L. interrogans* to breach the physical barriers of the host. This work identifies a mechanism by which an abundant but non-essential lipoprotein contributes to the cell mechanics of a pathogenic spirochete, establishing a paradigm where cell rigidity is an infection route-dependent virulence factor. The elucidation of this specialized structural function is vital for understanding the pathogenesis of leptospirosis. Given its role in tissue invasion, LipL32 may represent a potential target for strategies aimed at interfering with early stages of infection. For example, disrupting the dense packing or periplasmic organization of LipL32 could impair the mechanical properties required for efficient penetration of host tissues. While such approaches may not directly affect bacterial viability or later stages of infection, they could potentially complement conventional antimicrobial strategies.

## Methods

### Leptospira strains

*L. interrogans* serovar Manilae strain UP-MMC-NIID (wild-type, WT)^15,38,39^, the transposon insertion mutant *lipL32::*Tn (ΔLipL32), its complemented strain, and the WT strain carrying pNKLiG1 (vector control)^37^ were used in this study. The ΔLipL32 mutant was isolated from a random *Himar1* transposon mutant library generated in the WT strain via conjugation with *E. coli* β2163 harboring pCjTKS2^40^. The transposon insertion site was identified by semi-random PCR^41^, revealing insertion between bp 85 and 86 of the 819-bp *lipL32* gene. The *lipL32*-complemented ΔLipL32 strain was constructed as follows. The *lipL32* gene, including its putative promoter region, was amplified from genomic DNA of the WT strain using PrimeSTAR GXL DNA polymerase (Takara Bio, Japan) with primers 5′-AAGCTTATCGATACCGTCGAGAACAAGAAAGAGTCAGAGAA-3′ and 5′-GCGAGGCTGGCCGGCGTCGATTACTTAGTCGCGTCAGAAGC-3′. The PCR product was assembled into *Sal*I-digested pNKLiG1 using the NEBuilder HiFi DNA Assembly kit (New England Biolabs, USA) and propagated in *E. coli* π1^42^. The resulting plasmid was transformed into *E. coli* β2163 and subsequently introduced into the *lipL32::*Tn strain via conjugation. *Leptospira* strains were cultured in liquid Ellinghausen–McCullough–Johnson–Harris (EMJH) medium^43^ at 30 °C. The complemented and vector control strains were grown in EMJH medium supplemented with 25 µg/mL spectinomycin.

### Cryo-electron microscopy

Five milliliters of bacterial cultures were centrifuged at 1000 × *g* for 5 min to concentrate the cells to a final volume of 50–100 µL. Sample grids were prepared using a Vitrobot Mark IV (Thermo Fisher Scientific) maintained at 4 °C and 100% humidity. A 2.5-µL aliquot of the sample was loaded onto glow-discharged holey carbon grids (Quantifoil R1.2/1.3, Au 300 mesh). After 2 s of blotting with filter paper, the grids were instantly plunge-frozen into liquid ethane. Frozen grids were imaged using a Talos Arctica microscope (Thermo Fisher Scientific) operated at 200 kV and equipped with a Falcon 3 direct electron detector (Thermo Fisher Scientific). Dose-fractionated images were collected at nominal magnification of 92,000 × *g*, corresponding to a pixel size of 1.6 Å, with a total dose of 50 e^−^/Å² and defocus values ranging from −0.6 to −1.4 μm.

All data processing was performed using RELION 5^44^. Movie frames were motion-corrected in RELION, and CTF parameters were estimated using CTFFIND4^45^. Particle picking was performed manually by tracing the membrane, which was treated as helical segments for particle extraction using a box size of 640 pixels with an inter-box distance of 150 Å. Approximately 4000 particles were extracted and subjected to iterative 2D classification, each into 100 classes. Particles corresponding to segments in which the membrane was straight or convex toward the extracellular side were selected, and 2D classification was repeated until approximately 2500 particles remained.

### Expression and localization of LipL32

For preparation of whole-cell lysates, 1.5 × 10^8^ leptospiral cells were suspended in SDS-PAGE sample buffer and boiled at 100 °C for 8 min. The lysates were fractionated on 5–20% SDS-PAGE gels and visualized by Coomassie Brilliant Blue (CBB) staining using EzStain Aqua (AE-1340; ATTO Corporation). To examine the cellular localization of LipL32 in *L. interrogans* strains, Triton ×-114 phase partitioning was performed according to the method of Zuerner et al.^46^, with the following modifications. Ten milliliters of *Leptospira* cultures were centrifuged at 4000 × *g* for 20 min at 4 °C, and the resulting pellets were washed with Tris-buffered saline (TBS; 20 mM Tris-HCl [pH 7.5], 150 mM NaCl). The cell pellets were resuspended in 0.7 mL TBS containing 2% Triton ×-114 and a protease inhibitor cocktail (Roche), gently mixed on a rotator for 1 h at 4 °C, and centrifuged at 16,900 × *g* for 20 min at 4 °C. The resulting pellets were collected as the protoplasmic cylinder (PC) fraction. Subsequently, 78 μL of 100 mM CaCl₂ was added to the supernatant, which was incubated at 37 °C for 15 min and centrifuged at 2000 × *g* for 10 min at room temperature to separate the aqueous phase (PS fraction) from the detergent phase (OM fraction). Each phase was washed twice with TBS (detergent phase) or Triton ×-114 solution (aqueous phase). Proteins in each fraction were precipitated by adding 10 volumes of acetone, followed by centrifugation at ≥14,400 × *g* for 20 min at 4 °C. The resulting pellets were dissolved in SDS-PAGE sample buffer (120 μL for the PS and OM fractions and 300 μL for the PC fraction), and 10 μL of each sample was analyzed by 5–20% SDS–PAGE, followed by CBB staining.

### Growth assay

*Leptospira* cells grown in EMJH medium were diluted 1:10 into a fresh EMJH medium. EMJH media containing *Leptospira* cells were incubated at 30 °C, and their optical densities at 420 nm were measured.

### Morphology analysis using a dark-field microscope

*Leptospira* cells were harvested by centrifugation at 1000 × *g* for 10 min at room temperature. The bacterial precipitate was suspended in 20 mM potassium phosphate buffer (pH 7.4, PB) containing 100 µM carbonyl cyanide m-chlorophenylhydrazone (CCCP, FUJIFILM Wako Pure Chemical Corporation, Japan) (PB/CCCP), by which *Leptospira* cells were paralyzed^47^. The paralyzed cells were infused into a flow chamber and observed with a dark-field microscope (microscope body BX50, oil-immersion objective ×100 LMPlanFLN, ×5 relay lens, oil dark-field condenser, high-brightness light source U-LGPS, Olympus, Japan). The cell images were captured by a CMOS camera (ORCA-Spark, Hamamatsu Photonics, Japan), and the morphology of individual cells was analyzed using ImageJ.

### Stiffness measurement

*Leptospira* cells were paralyzed as performed for the morphology analysis (see above). The paralyzed cells were infused into a flow chamber and incubated for 1 h at room temperature. After flushing out floating cells with PB/CCCP, 1-μm polystyrene beads (ThermoFisher Scientific, Waltham, MA) diluted to 1:200 in PB/CCCP were infused into the flow chamber. The beads spontaneously attached to the bacterial surface without linkers^11^ were confirmed, and floating beads were removed with PB/CCCP.

Cell stiffness was measured by optical tweezers^48^. The optical-tweezers system (laser diode L1064H1, LD current controller LDC210C, Thorlabs Inc., Newton, NJ) was assembled into a bright-field microscope (microscope body IX70, oil-immersion objective ×100 LMPlanFLN, high-brightness light source U-LGPS, Olympus, Japan) and recorded using a CMOS camera (DFK33UX249, The Imaging Source, Germany) at a frame rate of 250 Hz. The optical tweezers trapped a bead adhering to a cell anchored to the glass. The cell body was bent by shifting the bead position using a piezoelectric stage. The bent cell body was recovered to its original shape by turning off the laser while resisting the viscoelastic force acting on the bead and cell body. The restoring force and drag force are given by ${kR}$ and $\gamma \dot{R}$ respectively, where k is the spring constant of the cell body, R is the bead position, and γ is the drag coefficient of the sum of cell body (${\gamma }_{{{\rm{C}}}}$) and bead (${\gamma }_{{{\rm{B}}}}$): ${\gamma }_{{{\rm{C}}}}=4\pi \mu L/\left[{\mathrm{ln}}\left(\frac{L}{2{r}_{{{\rm{C}}}}}\right)+0.84\right]$^49^ and ${\gamma }_{{{\rm{B}}}}=6\pi \mu {r}_{{{\rm{B}}}}$ (from the Stokes’ law), where L in the cell length (measured for individual cells), μ is the medium viscosity (0.9 mPa×s), ${r}_{{{\rm{C}}}}$ is the cell radius (0.07 μm), and ${r}_{{{\rm{B}}}}$ is the bead radius (0.5 μm). Assuming the balance between the restoring and drag forces yields $R=A\exp \left[(-k/\gamma )t\right]+C$ (see the Fig. 3 legend). Despite the introduction of a great approximation into the model, in agreement with this, tracking of the bead position after release from the trap gives a relaxation curve (Fig. 3c). The three-dimensional bead positions were traced by a custom-made image analysis program developed using Python (Supplementary Fig. 6).

### Agar-penetration assay

The bacterial density of the cultured *Leptospira* was adjusted to 1 × 10^8^ bacteria/mL (OD_420_ ~0.1) with EMJH medium. Agar (Bacto™ Agar) dissolved in PB was infused into a flow chamber and left at room temperature until solidified. The bacterial solution was infused into the chamber from the opposite side of the agar phase and incubated for 30 min at room temperature. The agar-liquid interface was recorded at a frame rate of 30 Hz, and the brightness of the area was analyzed with the ImageJ software.

### Motility assay

The *Leptospira* cells grown in EMJH were diluted 1:100 into PB and infused into a flow chamber. The bacteria were observed using a dark-field microscope (microscope body BX53, dry dark-field condenser, objective ×20 UPlan Fl, halogen lamp, Olympus, Japan) and recorded with a CMOS camera (ORCA-Fusion, Hamamatsu Photonics, Japan) at a frame rate of 100 Hz. Swimming speeds were measured by tracking individual cell centroids using ImageJ^50^. The cell-body rotation was quantified by measuring the time course of the hook-shaped cell end gyration, which was derived from the deformation (changes in curvature) of the hook in two-dimensional images^51^. Representative examples of the data analysis for swimming speed and rotation are provided in Supplementary Fig. 8a, b.

### Statistics and reproducibility

The exact sample sizes for each experiment are reported in the figure legends. Each experiment was independently repeated at least three times to ensure reproducibility. For cell-stiffness measurements and motility assays, the non-parametric, two-sided Mann–Whitney *U* test was used for statistical analysis, as these data did not follow a Gaussian distribution. For PG density, the statistical significance between group averages was assessed using Weighted Least Squares regression via the *statsmodels* library in Python, where the percentage of the total sample size for each average was used as the statistical weight. The exact *P*-values yielded by each statistical analysis are indicated in the figure legends.

### Reporting summary

Further information on research design is available in the Nature Portfolio Reporting Summary linked to this article.

## Supplementary information

Supplementary Information

Reporting Summary

## Data Availability

All data supporting the findings of this study are available on figshare (https://doi.org/10.6084/m9.figshare.32231823)^52^. All other data that support the findings of this study are available from the corresponding author upon reasonable request.

## Code availability

The custom Python scripts used for image processing and data analysis are available on GitHub at https://github.com/shuichi-nakamura-e8/Abe-Koizumi-Takazaki-et-al.-Communications-Biology-2026 and have been archived in Zenodo under https://doi.org/10.5281/zenodo.20134597^53^.

## References

[1] Sun, J., Rutherford, S. T., Silhavy, T. J. & Huang, K. C. Physical properties of the bacterial outer membrane. *Nat. Rev. Microbiol.* **20**, 236–248 (2022). 34732874 10.1038/s41579-021-00638-0 PMC8934262

[2] Nan, B. & Zusman, D. R. Novel mechanisms power bacterial gliding motility. *Mol. Microbiol.* **101**, 186–193 (2016). 27028358 10.1111/mmi.13389 PMC5008027

[3] Fivenson, E. M. et al. A role for the Gram-negative outer membrane in bacterial shape determination. *Proc. Natl. Acad. Sci. USA* **120**, e2301987120 (2023). 37607228 10.1073/pnas.2301987120 PMC10469335

[4] Deghelt, M. et al. Peptidoglycan–outer membrane attachment generates periplasmic pressure to prevent lysis in Gram-negative bacteria. *Nat. Microbiol.* **10**, 1963–1974 (2025). 40730909 10.1038/s41564-025-02058-9

[5] Nakamura, S. Spirochete flagella and motility. *Biomolecules* **10**, 550 (2020). 32260454 10.3390/biom10040550 PMC7225975

[6] Charon, N. W. et al. The unique paradigm of spirochete motility and chemotaxis. *Annu. Rev. Microbiol.* **66**, 349–370 (2012). 22994496 10.1146/annurev-micro-092611-150145 PMC3771095

[7] Brandt, M. E., Riley, B. S., Radolf, J. D. & Norgard, M. V. Immunogenic integral membrane proteins of *Borrelia burgdorferi* are lipoproteins. *Infect. Immun.* **58**, 983–991 (1990). 2318538 10.1128/iai.58.4.983-991.1990 PMC258571

[8] Guo, B. P., Brown, E. L., Dorward, D. W., Rosenberg, L. C. & Höök, M. Decorin-binding adhesins from *Borrelia burgdorferi*. *Mol. Microbiol.* **30**, 711–723 (1998). 10094620 10.1046/j.1365-2958.1998.01103.x

[9] Cameron, C. E. Identification of a *Treponema pallidum* laminin-binding protein. *Infect. Immun.* **71**, 2525–2533 (2003). 12704124 10.1128/IAI.71.5.2525-2533.2003 PMC153296

[10] Picardeau, M. Virulence of the zoonotic agent of leptospirosis: still terra incognita?. *Nat. Rev. Microbiol.* **15**, 297–307 (2017). 28260786 10.1038/nrmicro.2017.5

[11] Tahara, H. et al. The mechanism of two-phase motility in the spirochete *Leptospira*: Swimming and crawling. *Sci. Adv.* **4**, eaar7975 (2018). 29854948 10.1126/sciadv.aar7975 PMC5976277

[12] Adler, B. & de la Peña Moctezuma, A. *Leptospira* and leptospirosis. *Vet. Microbiol.* **140**, 287–296 (2010). 19345023 10.1016/j.vetmic.2009.03.012

[13] Costa, F. et al. Global morbidity and mortality of leptospirosis: a systematic review. *PLoS Negl. Trop. Dis.* **9**, e0003898 (2015). 26379143 10.1371/journal.pntd.0003898 PMC4574773

[14] Haake, D. A. et al. Leptospiral outer membrane proteins OmpL1 and LipL41 exhibit synergistic immunoprotection. *Infect. Immun.* **67**, 6572–6582 (1999). 10569777 10.1128/iai.67.12.6572-6582.1999 PMC97069

[15] Koizumi, N. & Watanabe, H. Leptospiral immunoglobulin-like proteins elicit protective immunity. *Vaccine* **22**, 1545–1552 (2004). 15063580 10.1016/j.vaccine.2003.10.007

[16] Cullen, P. A., Cordwell, S. J., Bulach, D. M., Haake, D. A. & Adler, B. Global analysis of outer membrane proteins from *Leptospira interrogans* serovar Lai. *Infect. Immun.* **70**, 2311–2318 (2002). 11953365 10.1128/IAI.70.5.2311-2318.2002 PMC127947

[17] Pinne, M. & Haake, D. A. LipL32 is a subsurface lipoprotein of *Leptospira interrogans*: presentation of new data and reevaluation of previous studies. *PLoS ONE* **8**, e51025 (2013). 23323152 10.1371/journal.pone.0051025 PMC3544172

[18] Lucas, D. S. D. et al. Recombinant LipL32 and LigA from *Leptospira* are unable to stimulate protective immunity against leptospirosis in the hamster model. *Vaccine* **29**, 3413–3418 (2011). 21396409 10.1016/j.vaccine.2011.02.084

[19] Murray, G. L. et al. Major surface protein LipL32 is not required for either acute or chronic infection with *Leptospira interrogans*. *Infect. Immun.* **77**, 952–958 (2009). 19103763 10.1128/IAI.01370-08 PMC2643616

[20] Feng, X., Li, G.-Y., Ramier, A., Eltony, A. M. & Yun, S.-H. In vivo stiffness measurement of epidermis, dermis, and hypodermis using broadband Rayleigh-wave optical coherence elastography. *Acta Biomater.* **146**, 295–305 (2022). 35470076 10.1016/j.actbio.2022.04.030 PMC11878153

[21] Guo, Z. et al. Fabrication of hydrogel with cell adhesive micropatterns for mimicking the oriented tumor-associated extracellular matrix. *ACS Appl. Mater. Interfaces* **6**, 10963–10968 (2014). 24989081 10.1021/am5023946

[22] Jayawardena, I. et al. Evaluation of techniques used for visualisation of hydrogel morphology and determination of pore size distributions. 10.1039/D2MA00932C (2023).

[23] Maaloum, M., Pernodet, N. & Tinland, B. Agarose gel structure using atomic force microscopy: gel concentration and ionic strength effects. *Electrophoresis* **19**, 1606–1610 (1998). 9719534 10.1002/elps.1150191015

[24] Raddi, G. et al. Three-dimensional structures of pathogenic and saprophytic *Leptospira* species revealed by cryo-electron tomography. *J. Bacteriol.* **194**, 1299–1306 (2012). 22228733 10.1128/JB.06474-11 PMC3294836

[25] Ratet, G. et al. LipL21 lipoprotein binding to peptidoglycan enables Leptospira interrogans to escape NOD1 and NOD2 recognition. *PLoS Pathog.* **13**, e1006725 (2017). 29211798 10.1371/journal.ppat.1006725 PMC5764436

[26] Loskill, P. et al. Reduction of the peptidoglycan crosslinking causes a decrease in stiffness of the *Staphylococcus aureus* cell envelope. *Biophys. J.* **107**, 1082–1089 (2014). 25185544 10.1016/j.bpj.2014.07.029 PMC4156677

[27] Lindemann, C. B. & Lesich, K. A. The mechanics of cilia and flagella: what we know and what we need to know. *Cytoskeleton* **81**, 648–668 (2024). 38780123 10.1002/cm.21879

[28] Ghanaeian, A. et al. Integrated modeling of the Nexin-dynein regulatory complex reveals its regulatory mechanism. *Nat. Commun.* **14**, 5741 (2023). 37714832 10.1038/s41467-023-41480-7 PMC10504270

[29] Masters, E. A. et al. Identification of penicillin binding protein 4 (PBP4) as a critical factor for *Staphylococcus aureus* bone invasion during osteomyelitis in mice. *PLoS Pathog.* **16**, e1008988 (2020). 33091079 10.1371/journal.ppat.1008988 PMC7608983

[30] Zhang, Y. et al. Multiple stiffening effects of nanoscale knobs on human red blood cells infected with *Plasmodium falciparum* malaria parasite. *Proc. Natl. Acad. Sci. USA* **112**, 6068–6073 (2015). 25918423 10.1073/pnas.1505584112 PMC4434686

[31] El Rayes, J., Rodríguez-Alonso, R. & Collet, J.-F. Lipoproteins in Gram-negative bacteria: new insights into their biogenesis, subcellular targeting and functional roles. *Curr. Opin. Microbiol.* **61**, 25–34 (2021). 33667939 10.1016/j.mib.2021.02.003

[32] Cohen, E. J., Ferreira, J. L., Ladinsky, M. S., Beeby, M. & Hughes, K. T. Nanoscale-length control of the flagellar driveshaft requires hitting the tethered outer membrane. *Science* **356**, 197–200 (2017). 28408605 10.1126/science.aam6512 PMC5963725

[33] Cascales, E., Bernadac, A., Gavioli, M., Lazzaroni, J.-C. & Lloubes, R. Pal lipoprotein of *Escherichia coli* plays a major role in outer membrane integrity. *J. Bacteriol.* **184**, 754–759 (2002). 11790745 10.1128/JB.184.3.754-759.2002 PMC139529

[34] Mathelié-Guinlet, M., Asmar, A. T., Collet, J.-F. & Dufrêne, Y. F. Lipoprotein Lpp regulates the mechanical properties of the *E. coli* cell envelope. *Nat. Commun.* **11**, 1789 (2020). 32286264 10.1038/s41467-020-15489-1 PMC7156740

[35] Rojas, E. R. et al. The outer membrane is an essential load-bearing element in Gram-negative bacteria. *Nature* **559**, 617–621 (2018). 30022160 10.1038/s41586-018-0344-3 PMC6089221

[36] Nakamura, S., Leshansky, A., Magariyama, Y., Namba, K. & Kudo, S. Direct measurement of helical cell motion of the spirochete *Leptospira*. *Biophys. J.* **106**, 47–54 (2014). 24411236 10.1016/j.bpj.2013.11.1118 PMC3907252

[37] Xu, J., Koizumi, N. & Nakamura, S. Crawling motility on the host tissue surfaces is associated with the pathogenicity of the zoonotic spirochete *Leptospira*. *Front. Microbiol.* **11**, 1886 (2020). 32849465 10.3389/fmicb.2020.01886 PMC7419657

[38] Fujita, R. et al. Comparison of bacterial burden and cytokine gene expression in golden hamsters in early phase of infection with two different strains of *Leptospira interrogans*. *PLoS ONE* **10**, e0132694 (2015). 26146835 10.1371/journal.pone.0132694 PMC4492770

[39] Tomizawa, R., Sugiyama, H., Sato, R., Ohnishi, M. & Koizumi, N. Male-specific pulmonary hemorrhage and cytokine gene expression in golden hamster in early-phase *Leptospira interrogans* serovar Hebdomadis infection. *Microb. Pathog.* **111**, 33–40 (2017). 28811249 10.1016/j.micpath.2017.08.016

[40] Abe, K., Koizumi, N. & Nakamura, S. Machine learning-based motion tracking reveals an inverse correlation between adhesivity and surface motility of the leptospirosis spirochete. *Nat. Commun.* **14**, 7703 (2023). 38052837 10.1038/s41467-023-43366-0 PMC10697978

[41] Slamti, L. & Picardeau, M. Construction of a library of random mutants in the spirochete *Leptospira biflexa* using a *mariner* transposon. *Methods Mol. Biol.* **859**, 169–176 (2012). 22367871 10.1007/978-1-61779-603-6_9

[42] Demarre, G. et al. A new family of mobilizable suicide plasmids based on broad host range R388 plasmid (IncW) and RP4 plasmid (IncPα) conjugative machineries and their cognate *Escherichia coli* host strains. *Res. Microbiol.* **156**, 245–255 (2005). 15748991 10.1016/j.resmic.2004.09.007

[43] Nakamura, S. Measurement of the cell-body rotation of *Leptospira*. *Methods Mol. Biol.* **2134**, 139–148 (2020). 32632866 10.1007/978-1-0716-0459-5_13

[44] Burt, A. et al. An image processing pipeline for electron cryo-tomography in RELION-5. *FEBS Open Bio.* **14**, 1788–1804 (2024). 39147729 10.1002/2211-5463.13873 PMC11532982

[45] Rohou, A. & Grigorieff, N. CTFFIND4: fast and accurate defocus estimation from electron micrographs. *J. Struct. Biol.* **192**, 216–221 (2015). 26278980 10.1016/j.jsb.2015.08.008 PMC6760662

[46] Zuerner, R. L., Knudtson, W., Bolin, C. A. & Trueba, G. Characterization of outer membrane and secreted proteins of *Leptospira interrogans* serovar *pomona*. *Microb. Pathog.* **10**, 311–322 (1991). 1895930 10.1016/0882-4010(91)90014-2

[47] Islam, M. S., Morimoto, Y. V., Kudo, S. & Nakamura, S. H^+^ and Na^+^ are involved in flagellar rotation of the spirochete *Leptospira*. *Biochem. Biophys. Res. Commun.* **466**, 196–200 (2015). 26348776 10.1016/j.bbrc.2015.09.004

[48] Abe, K., Kuribayashi, T., Takabe, K. & Nakamura, S. Implications of back-and-forth motion and powerful propulsion for spirochetal invasion. *Sci. Rep.* **10**, 13937 (2020). 32811890 10.1038/s41598-020-70897-z PMC7434897

[49] Tirado, M. M. & de la Torre, J. G. Translational friction coefficients of rigid, symmetric top macromolecules. Application to circular cylinders. *J. Chem. Phys.* **71**, 2581–2587 (1979).

[50] Nakamura, S. & Islam, Md. S. Motility of spirochetes. In *The Bacterial Flagellum: Methods and Protocols* (eds Minamino, T. & Namba, K.) 243–251 (Springer, New York, NY, 2017).

[51] Nakamura, S. Measurement of the cell-body rotation of *Leptospira*. In *Leptospira spp.: Methods and Protocols* (eds Koizumi, N. & Picardeau, M.) 139–148 (Springer US, New York, NY, 2020). 10.1007/978-1-0716-0459-5_13 32632866

[52] Data set for ‘A periplasmic layer formed by an outer membrane lipoprotein governs the cell-envelope integrity and stiffness of *Leptospira interrogans*’. *figshare* https://figshare.com/account/articles/32231823. 10.1038/s42003-026-10443-1 PMC13547282 42243461

[53] shuichi-nakamura-e8. shuichi-nakamura-e8/Abe-Koizumi-Takazaki-et-al.-Communications-Biology-2026: Initial release for manuscript. *Zenodo* 10.5281/ZENODO.20134597 (2026).

