# Supplementary material for: A periplasmic layer formed by an outer membrane lipoprotein governs the cell-envelope integrity and stiffness of Leptospira interrogans
Source: Commun Biol. 2026 Jun 4;9:1175. doi: 10.1038/s42003-026-10443-1 (PMC13547282; doi:10.1038/s42003-026-10443-1)
Supplement: Supplementary file 1 [file 42003_2026_10443_MOESM1_ESM.pdf]

Supplementary Information

A periplasmic layer formed by an outer membrane lipoprotein governs the cell-envelope integrity and stiffness of *Leptospira interrogans*

Abe K.[+], Koizumi N.[+], Takazaki H.[+], Hirose M., Takabe K., Kato T., and Nakamura S*.

[+]These authors contributed equally

*Correspondence: shuichi.nakamura.e8@tohoku.ac.jp

Contents:

Supplementary Fig. 1. Expression and localization of LipL32.

Supplementary Fig. 2. Effect of ΔLipL32 on the morphology and growth.

Supplementary Fig. 3. Flowchart and results of 2D cluster analysis.

Supplementary Fig. 4. Effect of ΔLipL32 on the peptidoglycan layer (PG).

Supplementary Fig. 5. Variation in cell envelope structure.

Supplementary Fig. 6. 3D tracking of a bead attached to the *Leptospira* cell end

Supplementary Fig. 7. Brightness profiles around the liquid-agar interface.

Supplementary Fig. 8. Effect of ΔLipL32 on the *Leptospira* motility.

Expression and localization of LipL32 in *Leptospira interrogans* strains

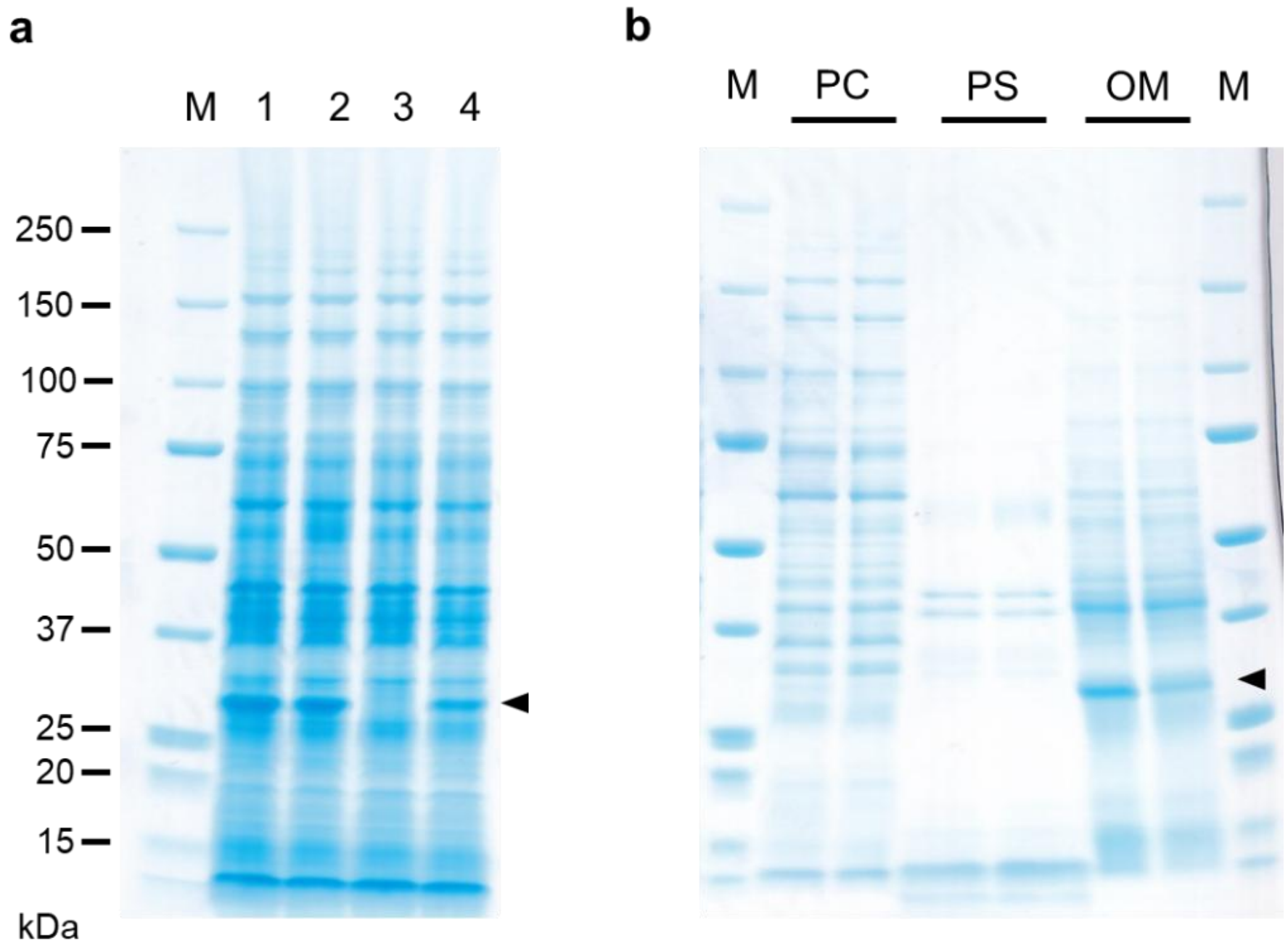

Supplementary Fig. 1. Expression and localization of LipL32. (a) Expression of LipL32 in *Leptospira interrogans* strain UP-MMC-NIID: wild type (WT, lane 1), WT carrying an empty vector (lane 2), *lipL32*::Tn (ΔLipL32) mutant (lane 3), and *lipL32*-complemented ΔLipL32 mutant (lane 4). (b) Cellular localization of LipL32 in WT (left) and ΔLipL32 mutant (right). PC, protoplasmic cylinder; PS, periplasmic space; OM, outer membrane fractions. M, molecular weight marker. LipL32 is indicated by arrowheads. Experiments were performed independently twice, and each preparation was analyzed by 5–20% SDS-PAGE; representative gels are shown.

Effect of ΔLipL32 on the morphology and growth

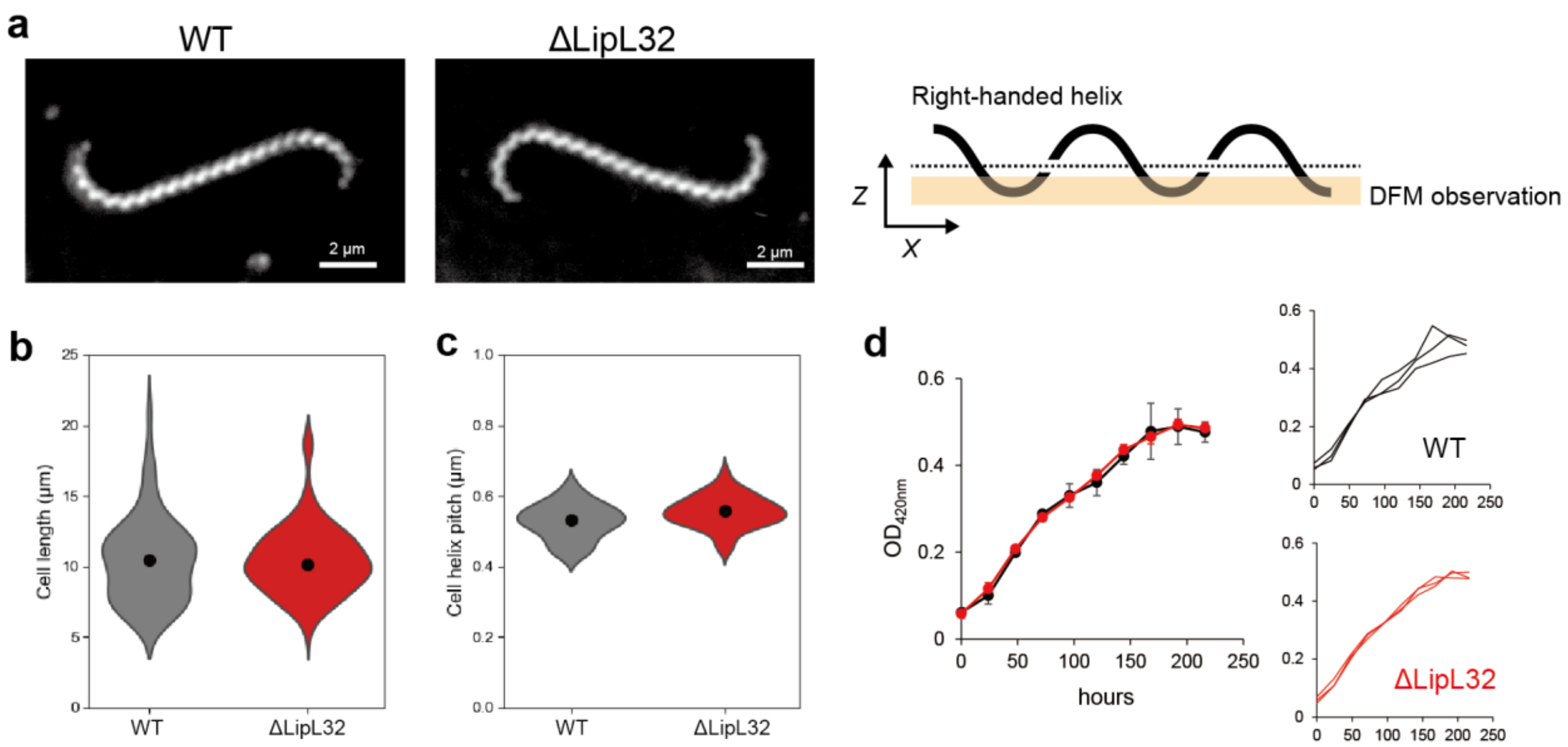

Supplementary Fig. 2. Effect of ΔLipL32 on the morphology and growth. (a) Dark-field micrographs of WT and ΔLipL32 mutant of *L. interrogans* (left panels). Both strains were grown in EMJH liquid medium without antibiotics. The right panel explains how a right-handed helix of the leptospiral cell body is visualized using a dark-field microscope equipped with a high numerical aperture (NA) objective. A tilt observed along the cell body of WT and ΔLipL32 mutant indicates that both strains have a right-handed helical cell body. (b) Cell length obtained by measuring 66 WT and 101 ΔLipL32 cells. (c) Cell helix pitch obtained by measuring 63 WT and 70 ΔLipL32 cells for cell helix pitch. Black dots represent the median of each population in *b* and *c*. (d) Growth curves. The left panel shows the mean values (dots with thick lines) and standard deviations (error bars) from three independent experiments (black for WT, red for ΔLipL32). Individual growth curves from each independent experiment are displayed as lines in the right panels.

2D cluster analysis of cryo-EM data

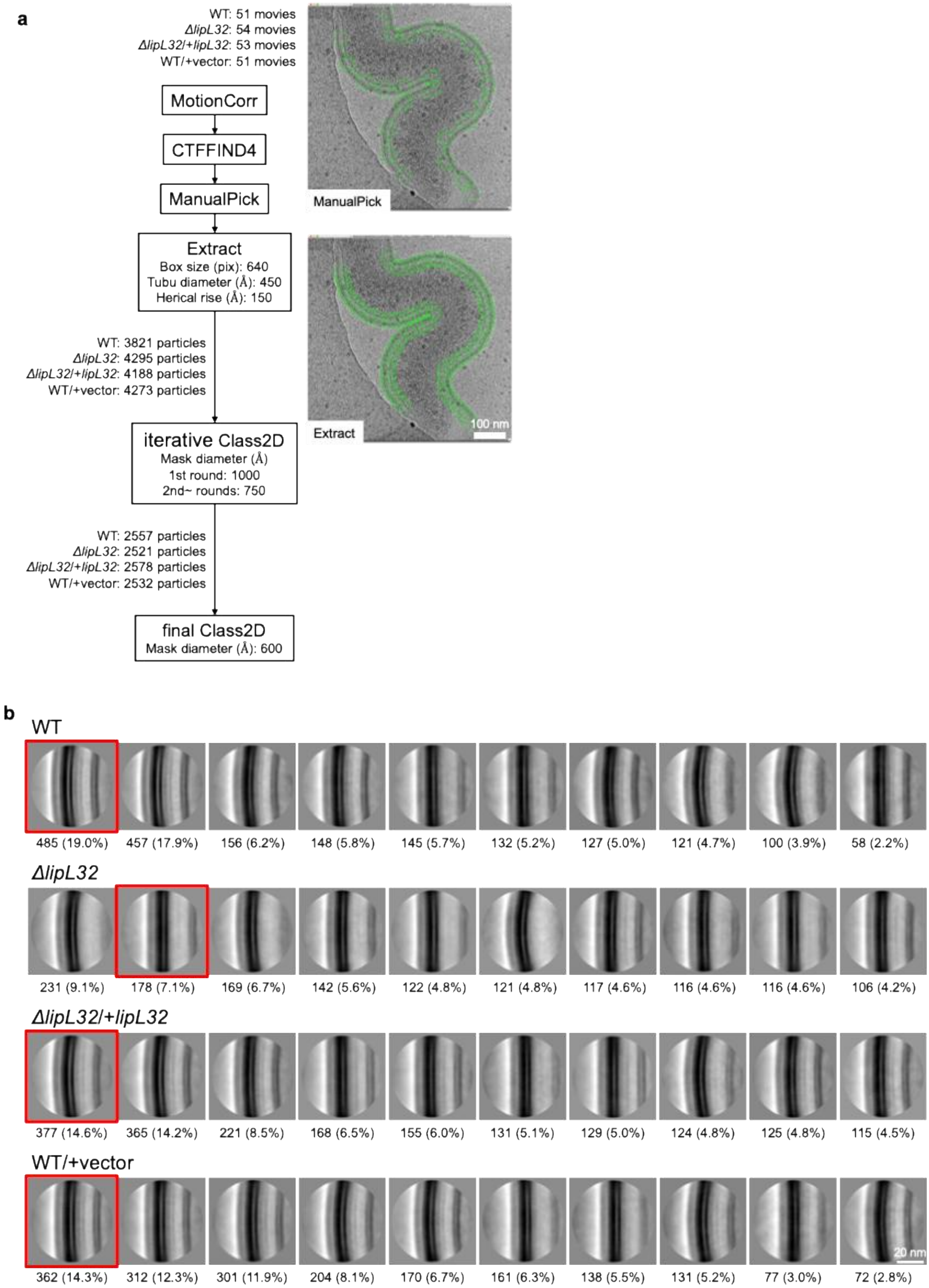

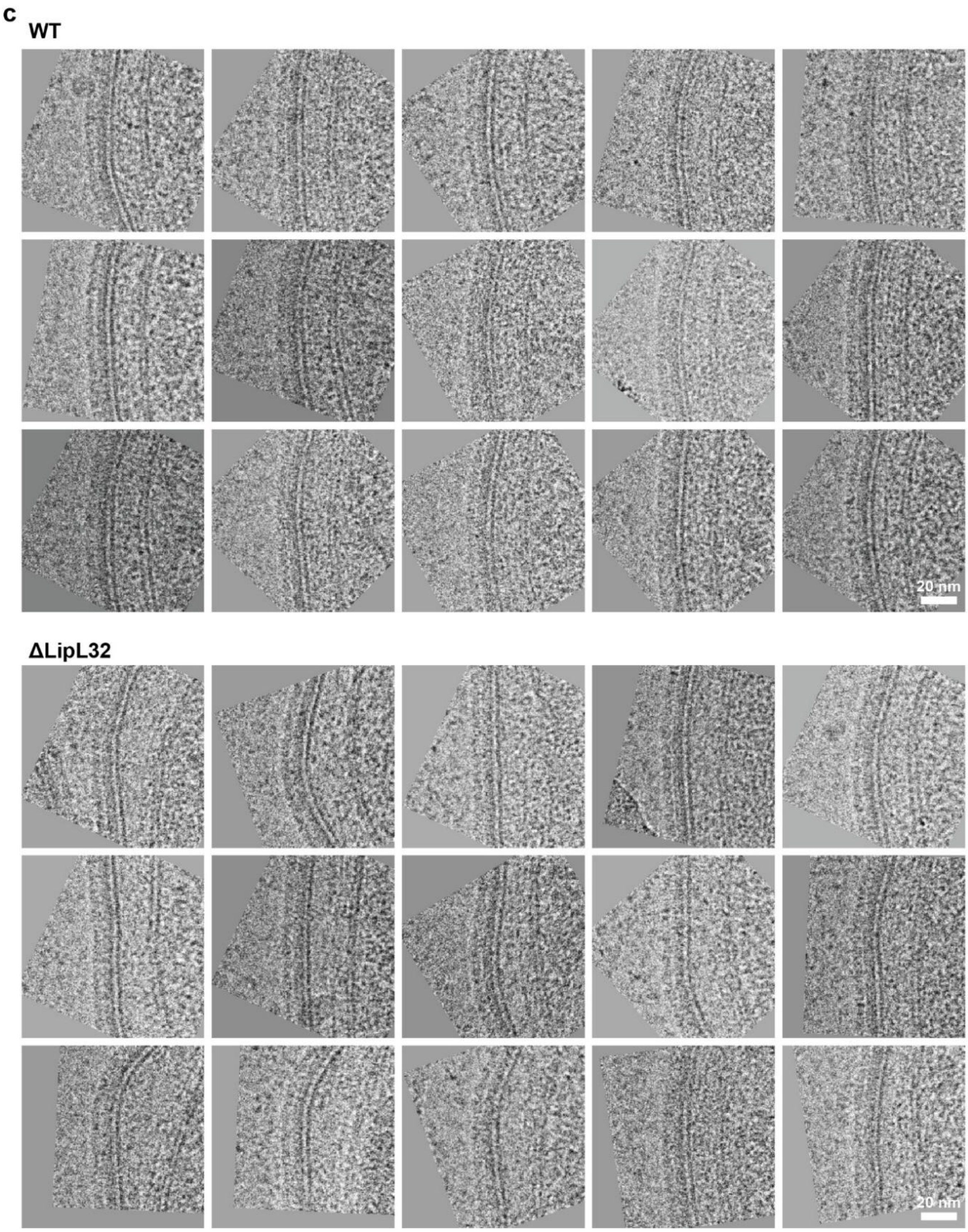

Supplementary Fig. 3. Flowchart and results of 2D classification. (a) Flowchart of the cryo-EM analysis. (b) Top 10 2D class averages with the largest number of particles. Numbers below each class average indicate the number of particles contributing to that average. Percentages in parentheses represent the fraction of all particles used in the final 2D classification, with the total set defined as 100%. Images indicated by red boxes correspond to the original images shown in Fig. 2b. (c) Gallery of representative raw cryo-EM images from the largest 2D classes shown in Supplementary Fig. 3b.

Effect of ΔLipL32 on the peptidoglycan layer

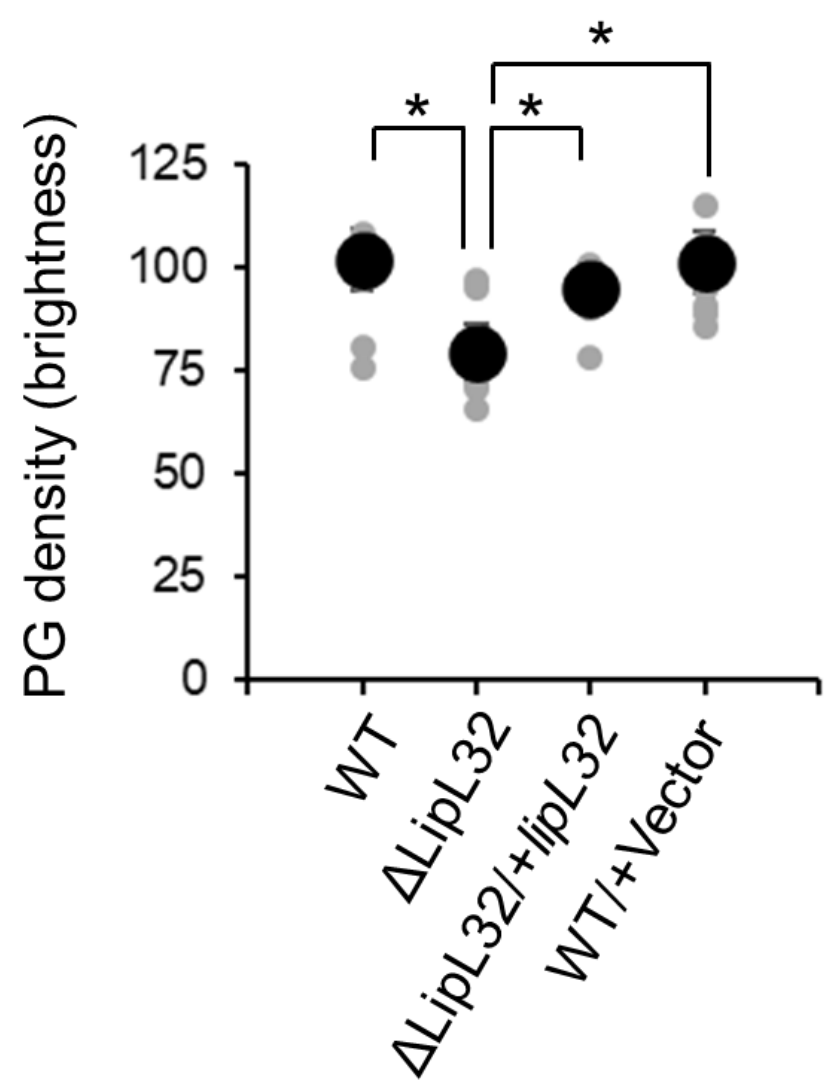

Supplementary Fig. 4. Effect of ΔLipL32 on the peptidoglycan layer (PG). The brightness of the PG observed in sets of averaged Cryo-EM images (Supplementary Fig. 3) was analyzed with ImageJ. The average values and standard deviations of the top 10 2D class averages (n = 10 independent class averages for each strain) are shown by black dots. Individual data from each 2D class average are shown as gray dots. The statistical significance between group averages was assessed by Weighted Least Squares (WLS) regression, considering the percentage of the total sample size used for each average as the statistical weight (*$P < 0.01$): WT vs ΔLipL32 $P = 6.48\times10^{-5}$, WT vs ΔLipL32/+lipL32 $P = 0.10$, WT vs vector control $P = 0.94$, ΔLipL32 vs ΔLipL32/+lipL32 $P = 2.81\times10^{-3}$, ΔLipL32 vs vector control $P = 9.09\times10^{-5}$, and ΔLipL32/+lipL32 vs vector control $P = 0.13$. Group comparisons were derived from the model coefficients. The analysis utilized the *statsmodels* library in Python.

Variation in cell envelope structure in the ΔLipL32 strain

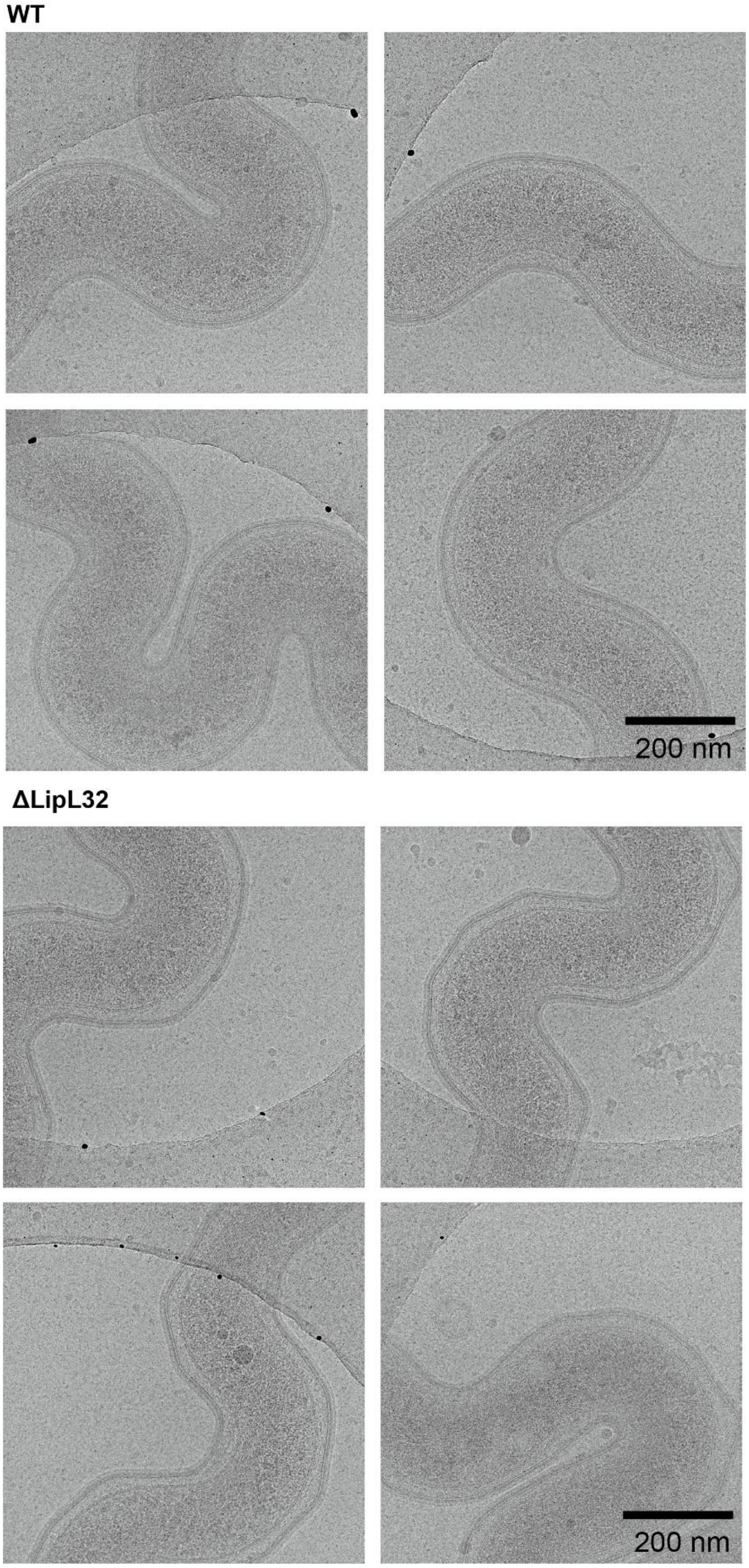

Supplementary Fig. 5. Representative cryo-EM micrographs of WT and ΔLipL32 cells (related to Fig. 2a). While WT cells exhibit a smooth and uniform outer membrane (OM), the ΔLipL32 mutant displays irregular OM morphology with significant structural diversity among individual cells.

3D tracking of a bead attached to a bacterial cell

In the present cell-stiffness measurement, the cell body was bent by trapping and moving a bead attached to the cell end, and the bead position after releasing from the laser trap was tracked. Cells partially attached to the glass surface were targeted by optical tweezers, and bead movements usually show a three-dimensional (3D) trajectory. Regarding the 3D tracking, we solved several problems as described below.

2D tracking. The simplest two-dimensional (2D) particle tracking uses a threshold for discriminating the "target" particle from the background. This method is available when the brightness distribution of the target particle is almost constant, ideally. However, when we observe the recovery of a bead attached to the *Leptospira* cell end at the fixed Z-axis position of a bright-field microscope, the bead image changes with the Z-axis movement (Supplementary Fig. 6a), making the threshold-dependent tracking difficult. To solve the problem, we first applied a machine-learning-based tracking, by which a dynamic foreground (a moving bead) is discriminated from a static background that is subtracted based on the Gaussian Mixture model[1]. The machine-learning method allows us to perform 2D tracking of a moving bead. However, it cannot determine the bead position immediately before being released from the laser, because the background subtraction can recognize the static object (i.e., a trapped bead) as a background. Since the original position is essential for analyzing the recovery rate of the bead, we applied the conventional method for particle detection using an arbitrary threshold. That is, we performed 2D tracking by using the "threshold" method and the "machine learning" method for determining the original position and tracking a moving bead, respectively.

Z-axis tracking. To determine the Z-axis position at each time, we prepared a set of bead images with known Z-axis displacement from the focal plane as a reference (Supplementary Fig. 6b). We determined the displacement in the Z direction by comparing the experimental data bead images, where the brightness distribution changes with displacement in the Z direction, with the reference based on the evaluation of the Structural Similarity Index Measure (SSIM) (Supplementary Fig. 6c). Integration of 2D tracking and Z-axis tracking results yields a three-dimensional trajectory (Supplementary Fig. 6d). Finally, the time-dependent displacement of the beads is determined from the three-dimensional trajectory, and the recovery rate is derived by fitting an exponential function (Supplementary Fig. 6e).

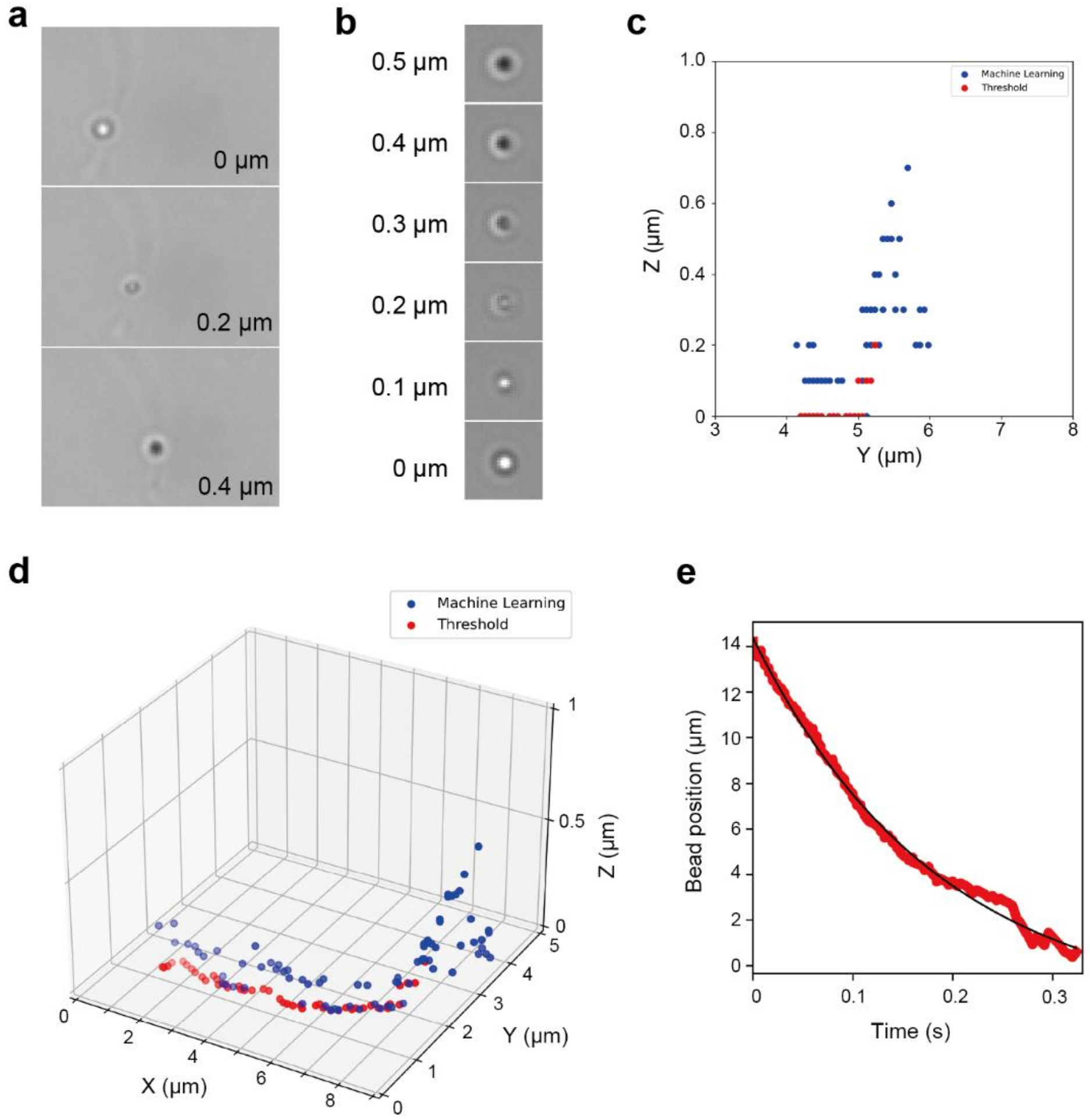

Supplementary Fig. 6. 3D tracking of a bead attached to the *Leptospira* cell end. (a) Change in the brightness distribution of a bead moving with the recovery of the cell body after being released from the laser. (b) Bead images with known Z-axis displacement from the focal plane (0 μm). (c) Z-axis movement of a bead. The red and blue dots represent the results obtained by the “threshold” method and “machine learning” method, respectively. (d) A 3D trajectory. (e) A recovery curve obtained from *d*.

Bacterial penetration into agar

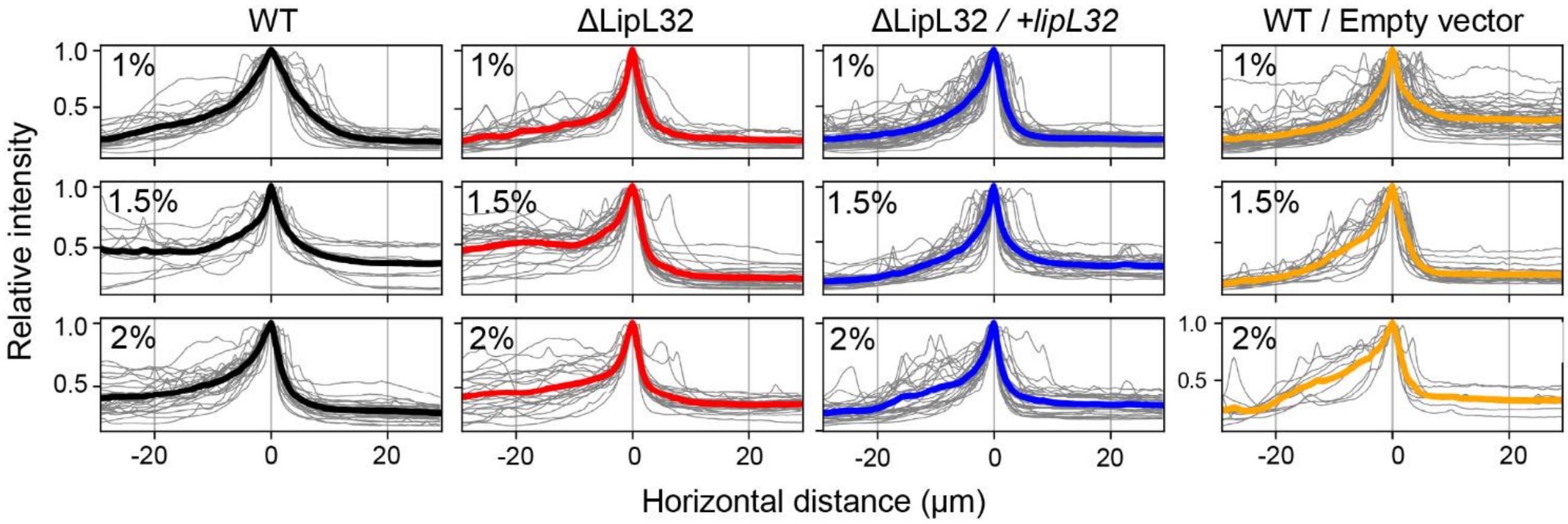

Supplementary Fig. 7. Brightness profiles around the liquid-agar interface. Brightnesses of the liquid-agar interface (0 of the horizontal axis) exposed with the WT, ΔLipL32, ΔLipL32*/lipL32* (gene-complemented strain), WT/+Empty vector (vector control) cells were measured. The data of WT and ΔLipL32 are the same as those shown in the main text. Thin lines represent individual profiles from independent measurements, and the thick line indicates the average of these profiles. See the main text for details of the experimental setup and analysis.

Effect of ΔLipL32 on the *Leptospira* motility

Regarding the insufficient recovery of gel-penetration observed in the *lipL32*-complemented strain and vector control, we assumed that genetic manipulations or antibiotic supplementation affected motility (see also *main text*). Motility is an essential virulence factor of *L. interrogans*[2,3]. The *Leptospira* motility form can be categorized into a “translational” mode, in which the cell is propelled forward by helical body rotation (Supplementary Fig. 8a), and a “rotation” mode, in which the cell rotates without net displacement (Supplementary Fig. 8b). Tracking of individual cells with distinguishing these two modes revealed that all strains exhibited similar levels of rotational activity (Supplementary Fig. 8c), indicating that flagellar function was not impaired. However, the swimming speeds of the complemented and vector control strains were significantly slower than those of the WT and ΔLipL32 cells (Supplementary Fig. 8d). Efficient swimming in spirochetes, including *Leptospira* spp., requires regulation of flagellar rotation at both cell ends, primarily mediated via chemotaxis signaling. Although the precise cause remains unclear, plasmid introduction or antibiotic selection may have affected intracellular signaling pathways to achieve smooth swimming.

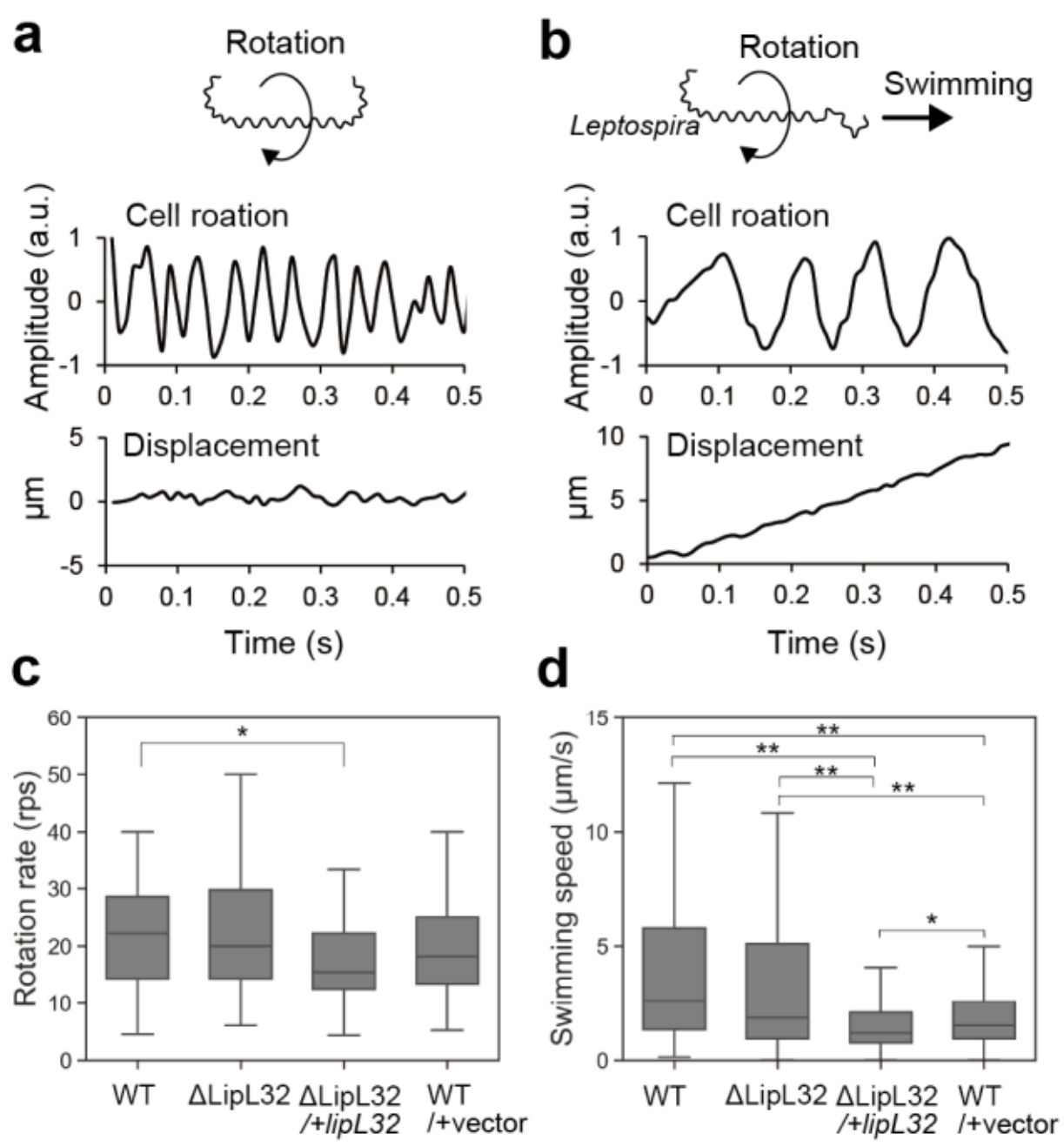

Supplementary Fig. 8. Effect of ΔLipL32 on the *Leptospira* motility. (a) A schematic of the rotation mode (top), and example data of cell-body rotation (middle) and displacement obtained by measuring a typical rotation-mode cell (bottom). (b) A schematic of the translation mode (top), and example data of cell-body rotation (middle) and displacement obtained by measuring a typical translation-mode cell (bottom). (c) Rotation rates of the cell body. 57 cells for WT, 60 cells for ΔLipL32, 63 cells for ΔLipL32 /+*lipL32*, and 63 cells for a vector control were measured. Box plots show the median (horizontal line in the box), the 25th and 75th percentiles (box), and the minimum and maximum values within 1.5 times the interquartile range (whiskers). Statistical analysis was performed by the two-sided Mann-

Whitney U test (*P < 0.05): WT vs ΔLipL32 P = 0.8956, WT vs ΔLipL32/+lipL32 P = 0.0076, WT vs vector control P = 0.2045, ΔLipL32 vs ΔLipL32/+lipL32 P = 0.0004, ΔLipL32 vs vector control P = 0.0482, and ΔLipL32/+lipL32 vs vector control P =0.1353. (d) Swimming speed. 145 cells for WT,138 cells for ΔLipL32, 148 cells for ΔLipL32 /+*lipL32*, and 169 cells for a vector control were measured. The box-and-whisker plots were made as in *c*. Statistical analysis was performed by the Mann-Whitney U test (*P < 0.05, **P < 0.01): WT vs ΔLipL32 P = 0.0898, WT vs ΔLipL32/+lipL32 $P = 0.0072\times10^{-6}$, WT vs vector control $P = 0.0071\times10^{-3}$, ΔLipL32 vs ΔLipL32/+lipL32 P = 0.0023, ΔLipL32 vs vector control P = 0.0003, and ΔLipL32/+lipL32 vs vector control P = 0.0184.

References

1. KaewTraKulPong, P. & Bowden, R. An improved adaptive background mixture model for real-time tracking with shadow detection. in *Video-Based Surveillance Systems: Computer Vision and Distributed Processing* (eds Remagnino, P., Jones, G. A., Paragios, N. & Regazzoni, C. S.) 135–144 (Springer US, Boston, MA, 2002). doi:10.1007/978-1-4615-0913-4_11.
2. Nakamura, S. Spirochete flagella and motility. *Biomolecules* **10**, 550 (2020).
3. Lambert, A. *et al.* FlaA proteins in *Leptospira interrogans* are essential for motility and virulence but are not required for formation of the flagellum sheath. *Infect. Immun.* **80**, 2019–2025 (2012).
