# Supplementary material for: A periplasmic layer formed by an outer membrane lipoprotein governs the cell-envelope integrity and stiffness of Leptospira interrogans
Source: Commun Biol. 2026 Jun 4;9:1175. doi: 10.1038/s42003-026-10443-1 (PMC13547282; doi:10.1038/s42003-026-10443-1)
Supplement: Supplementary file 2 [file 42003_2026_10443_MOESM2_ESM.pdf]

nature portfolio

Corresponding author(s): Shuichi Nakamura
Last updated by author(s): May 13, 2026

Reporting Summary

Nature Portfolio wishes to improve the reproducibility of the work that we publish. This form provides structure for consistency and transparency in reporting. For further information on Nature Portfolio policies, see our Editorial Policies and the Editorial Policy Checklist.

Statistics

For all statistical analyses, confirm that the following items are present in the figure legend, table legend, main text, or Methods section.

n/a	Confirmed	
☐	☒	The exact sample size (*n*) for each experimental group/condition, given as a discrete number and unit of measurement
☐	☒	A statement on whether measurements were taken from distinct samples or whether the same sample was measured repeatedly
☐	☒	The statistical test(s) used AND whether they are one- or two-sided<br>*Only common tests should be described solely by name; describe more complex techniques in the Methods section.*
☒	☐	A description of all covariates tested
☒	☐	A description of any assumptions or corrections, such as tests of normality and adjustment for multiple comparisons
☐	☒	A full description of the statistical parameters including central tendency (e.g. means) or other basic estimates (e.g. regression coefficient) AND variation (e.g. standard deviation) or associated estimates of uncertainty (e.g. confidence intervals)
☐	☒	For null hypothesis testing, the test statistic (e.g. *F*, *t*, *r*) with confidence intervals, effect sizes, degrees of freedom and *P* value noted<br>*Give P values as exact values whenever suitable.*
☒	☐	For Bayesian analysis, information on the choice of priors and Markov chain Monte Carlo settings
☒	☐	For hierarchical and complex designs, identification of the appropriate level for tests and full reporting of outcomes
☒	☐	Estimates of effect sizes (e.g. Cohen's *d*, Pearson's *r*), indicating how they were calculated

Our web collection on statistics for biologists contains articles on many of the points above.

Software and code

Policy information about availability of computer code

Data collection	IC Capture (version 2.5.1558.4007) and HCImageLive (version 4.8) were provided by The Imaging Source and Hamamatsu Photonics, respectively. EPU (version 2.12.1.2782) was used for dose fraction using Talos Arctica microscope equipped with a Falcon 3 direct electron detector (Thermo Fisher Scientific).
Data analysis	RELION5 (version 5.0.0), CTFFIND4 (version 4.1.13), ImageJ (version 1.54), Microsoft Excel (version 2601, build 16.0.19628.20214). Custom Python scripts used for specific data processing are available from the corresponding author upon reasonable request. These scripts utilize standard libraries including NumPy, Matplotlib, SciPy, Pandas, and OpenCV.

For manuscripts utilizing custom algorithms or software that are central to the research but not yet described in published literature, software must be made available to editors and reviewers. We strongly encourage code deposition in a community repository (e.g. GitHub). See the Nature Portfolio guidelines for submitting code & software for further information.

Data

Policy information about availability of data

All manuscripts must include a data availability statement. This statement should provide the following information, where applicable:

- Accession codes, unique identifiers, or web links for publicly available datasets
- A description of any restrictions on data availability
- For clinical datasets or third party data, please ensure that the statement adheres to our policy

All data supporting the findings of this study are available on figshare (DOI: 10.6084/m9.figshare.32231823)53. All other data that support the findings of this study are available from the corresponding author upon reasonable request.

Research involving human participants, their data, or biological material

Policy information about studies with human participants or human data. See also policy information about sex, gender (identity/presentation), and sexual orientation and race, ethnicity and racism.

Reporting on sex and gender	The study did not involve human participants.
Reporting on race, ethnicity, or other socially relevant groupings	The study did not involve human participants.
Population characteristics	The study did not involve human participants.
Recruitment	The study did not involve human participants.
Ethics oversight	The study did not involve human participants.

Note that full information on the approval of the study protocol must also be provided in the manuscript.

Field-specific reporting

Please select the one below that is the best fit for your research. If you are not sure, read the appropriate sections before making your selection.

☒ Life sciences ☐ Behavioural & social sciences ☐ Ecological, evolutionary & environmental sciences

For a reference copy of the document with all sections, see nature.com/documents/nr-reporting-summary-flat.pdf

Life sciences study design

All studies must disclose on these points even when the disclosure is negative.

Sample size	No statistical methods were used to predetermine sample size. Previous studies (Takabe et al., 2013, Microbiology and Immunology, 10.1111/1348-0421.12018, Takabe et al., 2017 Microbiology, 10.1099/mic.0.000420, and so on) have reported physical properties of bacteria based on n=20. Therefore, a lower limit of 20 bacteria was considered; however, to ensure the accuracy of the data, most experiments measured more than 50 bacteria, as described in the main text and Supplementary Information. EM data were sampled from more than 50 field of view for each grid.
Data exclusions	No data were excluded.
Replication	All attempts at replication were successful. Experiments and microscopic observations were independently repeated at least two times with similar results.
Randomization	In all experiments, bacteria were randomly sampled.
Blinding	Investigators were not blinded to allocation during experiments and outcome assessment as the physical phenotypes of the strains were being directly compared. However, strain identities were strictly tracked to prevent sample mix-ups.

Reporting for specific materials, systems and methods

We require information from authors about some types of materials, experimental systems and methods used in many studies. Here, indicate whether each material, system or method listed is relevant to your study. If you are not sure if a list item applies to your research, read the appropriate section before selecting a response.

Materials & experimental systems

n/a	Involved in the study
☒	☐ Antibodies
☒	☐ Eukaryotic cell lines
☒	☐ Palaeontology and archaeology
☒	☐ Animals and other organisms
☒	☐ Clinical data
☒	☐ Dual use research of concern
☒	☐ Plants

Methods

n/a	Involved in the study
☒	☐ ChIP-seq
☒	☐ Flow cytometry
☒	☐ MRI-based neuroimaging

Plants

Seed stocks	The study did not use plants.
Novel plant genotypes	The study did not use plants.
Authentication	The study did not use plants.
